# Protein- and RNA-Enhanced Fermentation by Gut Microbiota of the Earthworm Lumbricus terrestris

**DOI:** 10.1128/AEM.00657-18

**Published:** 2018-05-17

**Authors:** Lydia Zeibich, Oliver Schmidt, Harold L. Drake

**Affiliations:** a Department of Ecological Microbiology, University of Bayreuth, Bayreuth, Germany; University of Tennessee and Oak Ridge National Laboratory

**Keywords:** anaerobes, biopolymers, earthworm, fermentation, invertebrate microbiology

## Abstract

Earthworms are a dominant macrofauna in soil ecosystems and have determinative effects on soil fertility and plant growth. These invertebrates feed on ingested material, and gizzard-linked disruption of ingested fungal and bacterial cells is conceived to provide diverse biopolymers in the anoxic alimentary canals of earthworms. Fermentation in the gut is likely important to the utilization of ingested biopolymer-derived compounds by the earthworm. This study therefore examined the fermentative responses of gut content-associated microbes of the model earthworm Lumbricus terrestris to (i) microbial cell lysate (to simulate gizzard-disrupted cells) and (ii) dominant biopolymers of such biomass, protein, and RNA. The microbial cell lysate augmented the production of H_2_, CO_2_, and diverse fatty acids (e.g., formate, acetate, propionate, succinate, and butyrate) in anoxic gut content microcosms, indicating that the cell lysate triggered diverse fermentations. Protein and RNA also augmented diverse fermentations in anoxic microcosms of gut contents, each yielding a distinct product profile (e.g., RNA yielded H_2_ and succinate, whereas protein did not). The combined product profile of protein and RNA treatments was similar to that of cell lysate treatments, and 16S rRNA-based analyses indicated that many taxa that responded to cell lysate were similar to taxa that responded to protein or RNA. In particular, protein stimulated Peptostreptococcaceae, Clostridiaceae, and Fusobacteriaceae, whereas RNA stimulated Aeromonadaceae. These findings demonstrate the capacity of gut-associated obligate anaerobes and facultative aerobes to catalyze biopolymer-driven fermentations and highlight the potential importance of protein and RNA as substrates linked to the overall turnover dynamics of organic carbon in the alimentary canal of the earthworm.

**IMPORTANCE** The subsurface lifestyle of earthworms makes them an unnoticed component of the terrestrial biosphere. However, the propensity of these invertebrates to consume their home, i.e., soil and litter, has long-term impacts on soil fertility, plant growth, and the cycling of elements. The alimentary canals of earthworms can contain up to 500 ml anoxic gut content per square meter of soil, and ingested soil may contain 10^9^ or more microbial cells per gram dry weight, considerations that illustrate that enormous numbers of soil microbes are subject to anoxia during gut passage. Feeding introduces diverse sources of biopolymers to the gut, and the gut fermentation of biopolymers could be important to the transformation of matter by the earthworm and its capacity to utilize fermentation-derived fatty acids. Thus, this study examined the capacity of microbes in earthworm gut contents to ferment protein and RNA, dominant biopolymers of cells that become disrupted during gut passage.

## INTRODUCTION

Earthworms are a dominant macrofauna in soil ecosystems, and their feeding habits (i) result in substantial physical and chemical alterations of their habitats and (ii) have important effects on plant growth and the turnover of elements in the terrestrial biosphere (1–4). The alimentary canal of the earthworm constitutes an anoxic microzone in aerated soils (5), and diverse anaerobic activities in the gut are linked to the *in vivo* emission of nitrous oxide (N_2_O), dinitrogen (N_2_), molecular hydrogen (H_2_), and methane (CH_4_) by earthworms (6–11). The model earthworm Lumbricus terrestris has been used to assess gut-associated activities, and the anaerobic potentials of the gut community are exemplified by (i) the occurrence of over 30 mM fatty acids in the aqueous phase of the midgut and (ii) the marked capacity of gut-associated fatty acid-forming fermenters to consume diverse saccharides that are available in the gut (9, 12, 13). Fermentation is likely the dominant anaerobic process in the gut, with the *in situ* amount of reducing equivalents (i.e., electrons) in fermentation-derived fatty acids being over 1,000-fold greater than the *in situ* amount of reducing equivalents in the denitrification-derived gases N_2_O and N_2_ (8, 9). In this regard, fatty acids in the gut are utilized by the earthworm (14), illustrating that microbial fermentation in the gut constitutes a trophic link to the earthworm. Although these observations indicate that the earthworm gut is rich in anaerobic microbial activities, how these activities are potentially linked to the utilization of ingested biopolymers in the gut is largely unresolved.

Earthworms feed on biomass found in soil and litter, and ingested microbial cells are subject to rupture during passage through the gizzard, a hard muscular organ that abrasively grinds and disrupts ingested material, including microbial cells (6, 15). The gizzard disruption of microbial cells therefore introduces a wide range of nutrients into the alimentary canal, including protein, which, as the primary component of microbial cells, approximates 50% of microbial biomass on a dry weight basis (based on values from Saccharomyces cerevisiae and Escherichia coli [16]). Indeed, up to nearly 2 mM amino acids may occur in the aqueous phase of the earthworm gut (12), reinforcing the likelihood that protein hydrolysis in the gut yields amino acids that are subject to consumption during gut passage. In this regard, on the assumptions that (i) the cytoplasm of a microbial cell is 80% water and on a dry weight basis contains 50% protein and (ii) the average molecular weight of a representative amino acid in protein is 100, this amount of protein would theoretically yield 1 M polymeric amino acids in the cytoplasm of a gizzard-disrupted cell. As such, a microbial cell in this location of the alimentary canal (i.e., in the immediate vicinity of a ruptured cell) could experience a short-lived “tidal wave” of polypeptides. The availability of amino acids would be dependent on protein hydrolysis, and the secretion of proteases into the anterior of the alimentary canal including the gizzard indicates that the earthworm contributes to the breakdown of protein during gut passage (17). RNA is likewise a major component of microbial cells, constituting 6 to 20% of microbial biomass on a dry weight basis (16, 18), suggesting that the gizzard-facilitated rupture of microbes also yields RNA as an important biopolymer that is subject to consumption during gut passage.

These considerations indicate that microbes in the earthworm gut are provided with biopolymers derived from gizzard-disrupted cells, and the fermentation of these biopolymers by gut microbes might be important to the turnover dynamics of nutrients in the alimentary canal and the utilization of organic carbon by the earthworm. Thus, the hypotheses of the present study were that (i) the microbial community of the earthworm gut has the capacity to respond anaerobically to nutrient availability derived from disrupted microbial cells and (ii) protein and RNA, as primary biopolymers of disrupted cells, trigger fermentations that are facilitated by contrasting taxa. These hypotheses were addressed by determining (i) if microbial cell-free lysate, protein, or RNA enhances fermentation by gut-associated microbes of L. terrestris and (ii) which microbial taxa are engaged in the resulting fermentations.

## RESULTS

### Effect of cell lysate on gut fermentative taxa.

Diverse fermentations yield H_2_ and CO_2_ (19), and the anaerobic production of these gases can be considered an indicator of fermentation. In preliminary studies, fresh cell-free lysates of either S. cerevisiae or E. coli (used to simulate gizzard-disrupted microbial cells) augmented the anaerobic formation of H_2_ and CO_2_ (see Table S1 in the supplemental material), thus suggesting that a fermentative response to lysate was not dependent on the source of the lysate. Yeast-derived lysate was selected for more detailed studies because (i) larger microbial cells such as fungal cells are conceived to be more susceptible to rupture by the gizzard than smaller microbial cells (15, 20, 21) and (ii) the analysis of prokaryotic 16S rRNA would not be compromised (see Materials and Methods).

**FIG 1:**
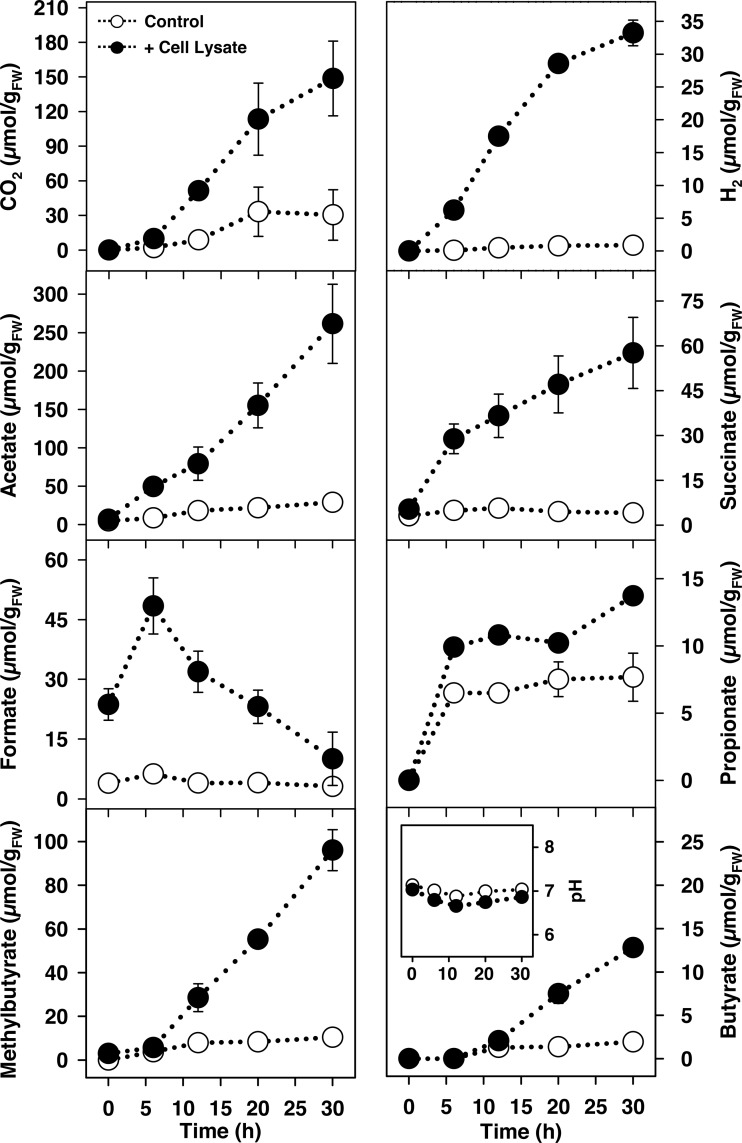
Effect of cell lysate on the fermentation product profiles of anoxic microcosms of L. terrestris gut contents. The amount of carbon derived from filter-sterilized lysate (6.0% dry weight) added per microcosm (10 ml containing 1 g_FW_ gut content) approximated 2.3 mmol. Controls lacked supplemental lysate (lysate alone did not display any fermentation activity). Values are the arithmetic average of three replicate analyses, and error bars indicate the standard deviations. Some standard deviations are smaller than the size of the symbol and therefore not apparent. The multiplication factor for converting the values into mM is 0.1 (e.g., 100 μmol/g_FW_ equals 10 mM). FW, fresh weight.

The rapid anaerobic formation of H_2_, CO_2_, and different fatty acids in yeast lysate treatments indicated that lysate triggered diverse fermentations (Fig. 1; see also Table S2 for statistical analyses of the dominant fermentation products). In contrast to the other fermentation products that accumulated, the formation of formate was transient, suggesting it was subject to consumption by secondary processes. The initial pH approximated 7 and was relatively stable (Fig. 1). The theoretical recoveries of lysate-derived carbon and reducing equivalents in fermentation products indicated that >50% of lysate-derived organic matter was converted to fermentation products (Table 1, lysate treatment).

**TABLE 1:** Estimated recoveries of carbon and reducing equivalents from anoxic microcosms of L. terrestris gut contents supplemented with S. cerevisiae lysate, protein, or RNA^*a*^

Product	Recoveries (%)^*b*^					
	Lysate treatment		Protein treatment		RNA treatment	
	Carbon	Reducing equivalents	Carbon	Reducing equivalents	Carbon	Reducing equivalents
CO_2_	5.2	NA^*c*^	3.1	NA	6.9	NA
H_2_	NA	0.7	NA	0.1	NA	3.1
Acetate	20.2	20.1	15.0	14.5	8.9	11.5
Methyl butyrate	18.1	23.4	13.8	17.4	—^*d*^	—
Succinate	9.0	7.8	—	—	4.7	5.3
Propionate	0.8	0.9	4.8	5.4	0.3	0.4
Butyrate	1.9	2.4	4.0	4.9	—	—
Formate	—	—	—	—	1.1	0.7
Lactate	0.1	0.1	—	—	1.7	2.2
Total	55.3	55.4	40.4	42.1	23.6 (44.9)^*e*^	23.2 (34.1)^*e*^

a See Fig. 1 for product profiles of lysate and Fig. 3 for product profiles of protein and RNA treatments. The method for calculating recoveries is provided in Materials and Methods.

b Recoveries were calculated at the end of the 30-h incubation and are based on the arithmetic average from three replicate analyses.

c NA, not applicable.

d —, no net increase of the product during the 30-h incubation.

e Parenthetical values are the estimated recoveries based on RNA-derived ribose as sole source of carbon and reducing equivalents.

Potential time-dependent shifts in the microbial community were assessed by 16S rRNA and 16S rRNA gene analyses. A total of 1,715,804 bacterial 16S rRNA and 16S rRNA gene sequences were obtained, and rarefaction analyses indicated that the most abundant taxa were targeted (see Fig. S1A). The numbers of detected and expected phylotypes as well as Shannon indices were lower at the end of the incubation period in lysate treatments than in unsupplemented controls (see Table S3).

**FIG 2:**
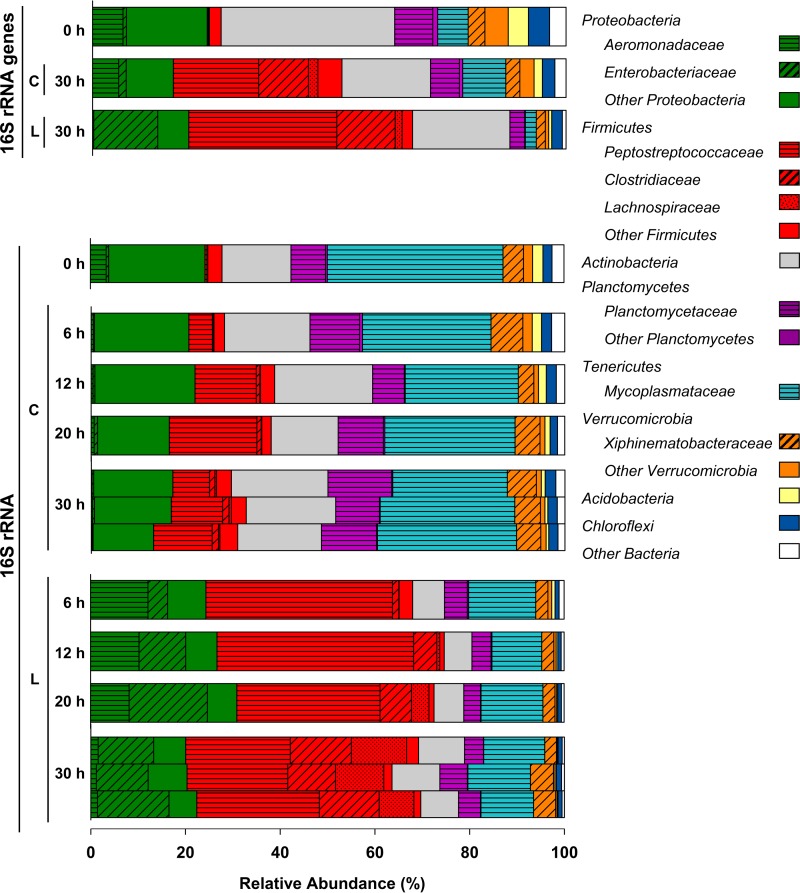
Effect of cell lysate on the temporal changes of the relative abundances of bacterial phyla in L. terrestris gut content microcosms based on the analyses of 16S rRNA and 16S rRNA genes. The most abundant families (families with ≥5% relative abundance in at least one sampling period) are displayed in the color of the respective phylum. Abbreviations: L, lysate treatment; C, unsupplemented control. Samples of the three replicates of a treatment were always pooled for each sampling time point, except for the 16S rRNA samples at the end of the 30-h incubation, in which each bar represents one replicate (the high similarity of the three replicates illustrates the reproducibility of the analyses). Process data are shown in Fig. 1 and information on all detected taxa is provided in “Sequence abundances” in Materials and Methods.

On the basis of the relative abundances of the detected 16S rRNA sequences in control and lysate treatments at the end of the incubation, the phylum Firmicutes was notably stimulated by lysate, with the associated families Peptostreptococcaceae, Clostridiaceae, and Lachnospiraceae displaying increased relative abundances in response to lysate (Fig. 2). The relative abundances of two families of the phylum Proteobacteria varied, with Aeromonadaceae-affiliated 16S rRNA sequences increasing initially in lysate treatments but decreasing with time and Enterobacteriaceae-affiliated 16S rRNA sequences increasing more gradually and dominating the Proteobacteria-affiliated sequences at the end of the incubation. The increases in relative abundances of Aeromonadaceae-, Clostridiaceae-, Enterobacteriaceae-, Lachnospiraceae-, and Peptostreptococcaceae-affiliated 16S rRNA sequences were supported by statistical analyses of the comparative relative abundances of the sequences in control and lysate treatments at the end of the incubation (see Table S4A). The stability of the pH during the incubation (Fig. 1) reinforced the likelihood that nutrient input rather than a change in pH was an important factor for the observed changes in the community composition of the lysate treatment. Mycoplasmataceae were represented by a phylotype with 99% similarity to “Candidatus Lumbricincola” sp. LR-B2, and 16S rRNA sequences of this phylotype had a high relative abundance in unsupplemented controls. Members of the genus “Candidatus Lumbricincola” were previously detected in tissues, gut contents, and casts of earthworms (22).

### Effects of protein and RNA on gut fermentative taxa.

As noted above, protein and RNA are major constituents of the microbial biomass. These two biopolymers were therefore examined to determine if they, like microbial cell lysate, triggered strong fermentative responses by gut content-associated microbes.

On the basis of the anaerobic production of H_2_, CO_2_, and fatty acids, initial assessments demonstrated that protein and RNA had strong effects on the fermentative activity of gut contents (see Fig. S2A and Table S5A). However, protein and RNA yielded dissimilar fermentation profiles. For example, large amounts of H_2_ accumulated in RNA treatments but did not accumulate in protein treatments. The rapid enhancement of fermentation by protein and RNA indicated that fermentative microbes in the gut contents were poised to respond to these biopolymers. Indeed, increasing amounts of protein and RNA yielded increasing amounts of CO_2_ and H_2_, respectively (see Table S6), (i) reinforcing the likelihood that fermentative microbes in the gut contents were not nutrient saturated and (ii) demonstrating a cause-and-effect relationship between fermentation and the availability of protein and RNA. To examine the potential specificity of this biopolymer-linked stimulation, gut contents were also challenged with cellulose and xylan, and the fermentative responses to these plant-derived polysaccharides were notably less than the fermentative responses to protein and RNA (Fig. S2B and Table S5B).

**FIG 3:**
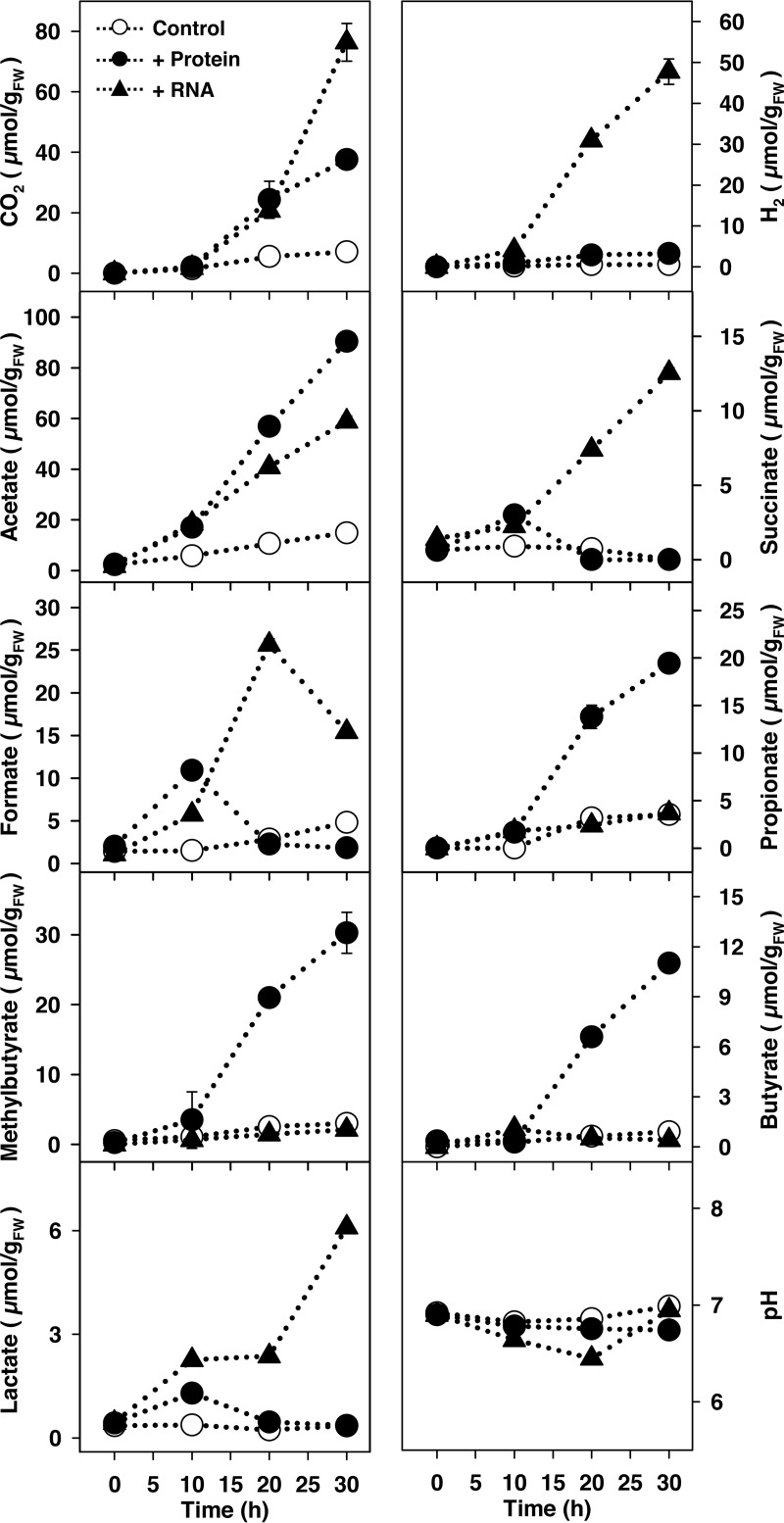
Effect of protein or RNA on the fermentation product profiles of anoxic microcosms of L. terrestris gut contents. The amounts of protein- and RNA-derived carbon added from filter-sterilized stock solutions approximated 1 mmol per microcosm (10 ml containing 1 g_FW_ gut content). Controls lacked supplemental protein or RNA (protein or RNA alone did not display any fermentation activity). Values are the arithmetic averages from three replicate analyses, and error bars indicate the standard deviations. Some standard deviations are smaller than the size of the symbol and therefore not apparent. The multiplication factor for converting the values into mM is 0.1 (e.g., 100 μmol/g_FW_ equals 10 mM). FW, fresh weight.

The marked capacity of gut-associated microbes to respond to protein and RNA prompted a more detailed analysis of the impact of these biopolymers on the gut microbial community. Time-resolved analyses of protein treatments revealed acetate, propionate, butyrate, and methyl butyrate as dominant fatty acids; in contrast, RNA treatments yielded formate, acetate, and succinate as the dominant fatty acids (Fig. 3; see also Table S2B and C for statistical analyses of the dominant fermentation products). As in the initial assessment above (Fig. S2A), H_2_ production was markedly more pronounced in RNA treatments (Fig. 3), demonstrating the reproducibility of this activity. However, we cannot exclude the possibility that H_2_ was also consumed. Formate was transient in both protein and RNA treatments, a pattern similar to that obtained in lysate treatments (Fig. 1). The initial pH approximated 7 and was relatively stable (Fig. 3). The combined product profile of the protein and RNA treatments was qualitatively very similar to the product profile of the lysate treatment.

Theoretical recoveries of carbon and reducing equivalents in the time-resolved protein and RNA treatments indicated that the supplemental amounts of these biopolymers were adequate for the observed fermentation products (Table 1, protein treatment and RNA treatment). Casamino Acids stimulated fermentation and yielded a similar product profile to that of the protein treatment (see Table S7), corroborating the capacity of gut content microbes to ferment amino acids. In this regard, the marked production of propionate and methyl butyrate in protein and Casamino Acids treatments is consistent with amino acid-linked fermentation (23–25). The hydrolysis of RNA yields ribose, purines, and pyrimidines. In this regard, (i) ribose stimulated fermentation (Table S7), (ii) the theoretical amounts of recovered carbon and redundant from supplemental RNA did not exceed the amounts available from RNA-derived ribose (Table 1, RNA treatments, parenthetical values), and (iii) the production of succinate and formate in RNA treatments is consistent with ribose-linked fermentation (26–28). These observations suggest that the dissimilation of RNA-derived ribose was likely important to RNA-coupled fermentation. Although adenine (a purine) and uracil (a pyrimidine) did not appear to appreciably enhance fermentation as single substrates (data not shown), we cannot exclude the possibility that RNA-derived purines and pyrimidines were utilized during RNA-based fermentation (e.g., were assimilated and used for cell biosynthesis, and thereby indirectly enhanced ribose-based fermentation). Ethanol was a major product in ribose treatments, constituting approximately 40% of the recovered reductant (Table S7). In contrast, ethanol was not detected in protein and Casamino Acids treatments. Ethanol and uracil had overlapping retention times on the high-performance liquid chromatograph column, which did not enable an accurate determination of ethanol in the RNA treatment. However, that ethanol was a major product of ribose fermentation is evidence that ethanol was formed during RNA-based fermentation. Ethanol is also produced during the earthworm gut content fermentation of xylose (13), confirming that ethanol is produced during pentose-based fermentations.

A total of 2,019,822 bacterial sequences were obtained, and rarefaction analyses indicated that the most abundant taxa were targeted (Fig. S2B). The numbers of detected and expected phylotypes decreased during incubation, a trend more pronounced for protein and RNA treatments than for unsupplemented controls, and Shannon indices decreased in protein and RNA treatments, whereas those in controls remained relatively constant (see Table S8). These results suggested that these supplemental biopolymers stimulated subgroups of the microbial community.

**FIG 4:**
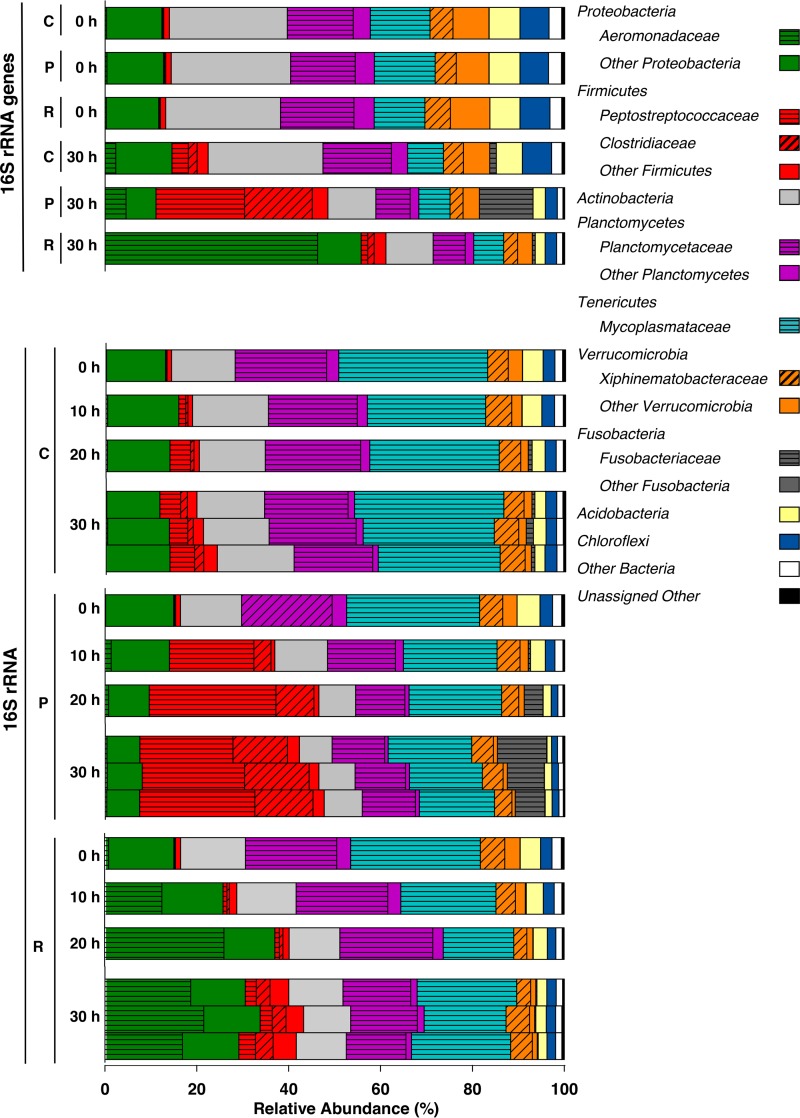
Effect of protein or RNA on the temporal changes of the relative abundances of bacterial phyla in L. terrestris gut content microcosms based on the analyses of 16S rRNA and 16S rRNA genes. The most abundant families (i.e., families with ≥5% relative abundance in at least one sampling period) are displayed in the color of the respective phylum. Abbreviations: P, protein treatment; R, RNA treatment; C, unsupplemented control. Samples of the three replicates of a treatment were always pooled for each sampling time point, except for the 16S rRNA samples at the end of the 30-h incubation, in which each bar represents one replicate (the high similarity of the three replicates illustrates the reproducibility of the analyses). Process data are shown in Fig. 3, and information on all detected taxa is provided in “Sequence abundances” in Materials and Methods.

The relative abundances of 16S rRNA gene sequences were very similar in all treatments prior to incubation; this was also the case for the relative abundances of 16S rRNA sequences (Fig. 4, sequences at 0 h). The 16S rRNA gene analyses indicated that Actinobacteria, Proteobacteria (Alphaproteobacteria and Gammaproteobacteria), Planctomycetes (Planctomycetaceae), Tenericutes (Mycoplasmataceae), and Verrucomicrobia (Xiphinematobacteriaceae) were abundant bacterial taxa in the gut contents prior to incubation, and the relative abundances of 16S rRNA sequences indicated that these taxa were active throughout the incubation period in unsupplemented controls (Fig. 4). Firmicutes-affiliated sequences displayed a modest increase in relative abundance in unsupplemented controls.

The marked change in relative abundances of 16S rRNA-based phylotypes in the first 10 h of incubation demonstrated that the bacterial community responded quickly to the availability of protein and RNA (Fig. 4). The relative abundances of 16S rRNA and 16S rRNA gene sequences indicated that Peptostreptococcaceae, Clostridiaceae, and Fusobacteriaceae were families that were stimulated in protein treatments, whereas Aeromonadaceae was the most abundant family that responded in the RNA treatment (Fig. 4). The increases in the relative abundances of Aeromonadaceae-, Clostridiaceae-, Fusobacteriaceae-, and Peptostreptococcaceae-affiliated 16S rRNA sequences were supported by statistical analyses of the comparative relative abundances of sequences in control and supplemental treatments at the end of the incubation (Table S4B). Members of the Mycoplasmataceae, Planctomycetaceae, and Xiphinematobacteriaceae families displayed relatively stable abundances in all treatments, suggesting that the subsistence of these families was not dependent on supplemental protein or RNA.

## DISCUSSION

Soil contains one of the most diverse microbiomes of the terrestrial biosphere, with a gram (dry weight) of soil having 10^9^ or more microbial cells (29, 30). L. terrestris is a model anecic earthworm, feeding on both soil and litter (31). Ingested material therefore delivers an enormous number of microorganisms to the oxygen-deficient digestive system of the earthworm. Ingestion coupled to the abrasive action of the gizzard introduces diverse biopolymers to the gut, and the marked stimulatory effect of protein and RNA, biopolymers that constitute primary components of gizzard-disrupted cells, demonstrated that fermentative microbes in gut contents were poised to respond to these biopolymers under anoxic conditions (Fig. 3).

### Fermentative microbes responsive to cell lysate, protein, and RNA.

The product profiles of cell lysate, protein, and RNA treatments indicated that these substrates were fermented by facultative aerobes and obligate anaerobes. It is noteworthy that H_2_ accumulated in RNA-based fermentations but did not accumulate in protein-based fermentation. Amino acid fermenters may engage non-H_2_-producing Stickland fermentations when H_2_ concentrations reach a certain level (32), which might partly explain why H_2_ did not accumulate to higher concentrations in protein treatments.

**FIG 5:**
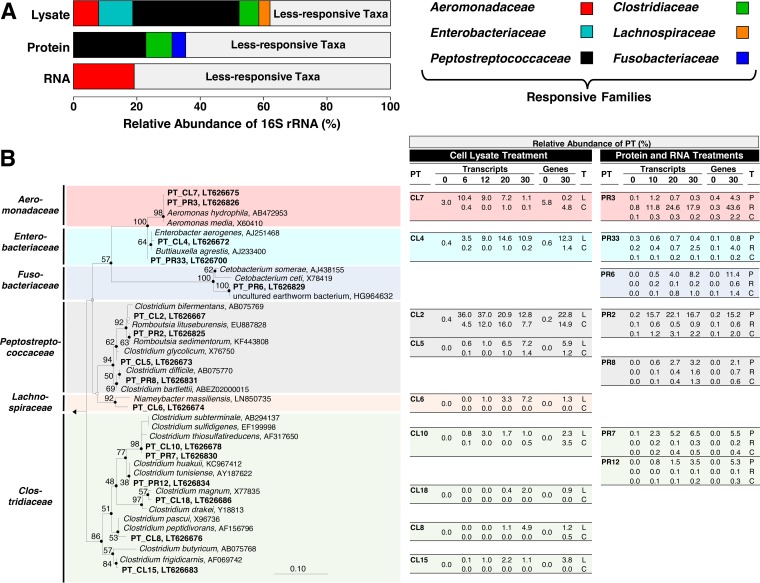
Average relative abundances of 16S rRNA sequences of the most responsive families of lysate, protein, and RNA treatments (A) and 16S rRNA-based phylogenetic tree of affiliated responsive phylotypes (in bold) and reference sequences (B). (A) Families were designated most responsive when a family in a given treatment displayed a minimum increase in relative abundance of 5% above the control values in at least one of the sampling periods. The values for each family are based on the arithmetic average from all abundances detected at 6, 12, 20, and 30 h for the cell lysate treatment and at 10, 20, and 30 h for the protein or RNA treatments. (B) Phylotypes (PT) are based on a sequence similarity cutoff of 97% and were designated responsive when a phylotype in a given treatment displayed a minimum increase in relative abundance of 2% above the control values in at least one of the sampling periods. The phylotypes are derived from the analysis of either 16S rRNA (designated as transcripts) or 16S rRNA genes (designated as genes). The phylogenetic tree was calculated using the neighbor-joining, maximum parsimony, and maximum likelihood methods. Solid circles at nodes indicate congruent nodes in three trees. Empty and gray circles at nodes indicate congruent nodes in two trees (neighbor-joining congruent with maximum parsimony or maximum parsimony congruent with maximum likelihood). Branch length and bootstrap values (1,000 resamplings) are from the maximum parsimony tree. The bar indicates 0.1 changes per nucleotide. Thermotoga maritima (AE000512) was used as the outgroup. Accession numbers are shown at the end of each branch. Relative abundances (in %) of phylotypes in the table are shown for each sampling period (i.e., 0, 6, 12, 20, and 30 h for the cell lysate treatment, and 0, 10, 20, and 30 h for the protein or RNA treatments). Closely related phylotypes (i.e., >97% sequence similarity) that increased in the cell lysate (L) treatment and protein (P) or RNA (R) treatments were placed on the same horizontal level. C, unsupplemented control, T, treatment.

The relative abundances of the most responsive taxa of the lysate treatment (i) constituted approximately 60% of the total abundance of the detected taxa and (ii) were greater than those of either the protein or RNA treatments (Fig. 5A). However, there was overlap between the responsive family-level taxa in lysate treatments and the responsive family-level taxa in the protein and RNA treatments, with the dominant responsive families of the protein and RNA treatments, i.e., Peptostreptococcaceae (protein), Clostridiaceae (protein), and Aeromonadaceae (RNA), collectively constituting approximately three-fourths of the responsive families in the lysate treatment (Fig. 5A). This trend extended to many phylotypes with a ≥97% nucleic acid sequence similarity (Fig. 5B) (e.g., CL2 [lysate] and PR2 [protein], and CL7 [lysate] and PR3 [RNA]). These findings support the likelihood that many of the responding taxa in the lysate treatment were responding to lysate-derived protein and RNA.

The phylotypes PR2, PR6, PR7, and PR12 displayed the strongest responses to protein. Of these four phylotypes, phylotype PR2, which was closely related to species of Romboutsia, was most responsive at both the transcript and gene levels (Fig. 5B). Obligate anaerobes of this genus occur in soil, humus, lake sediments, and the intestinal tracts of mammals (33–36). Romboutsia-affiliated species produce acetate, formate, ethanol, propionate, butyrate, isobutyrate, and methyl butyrate when fermenting amino acids or carbohydrates, and one species (Romboutsia lituseburensis [formerly Clostridium lituseburense], 99% identity to PR2) utilizes gelatin, chopped meat, and casein, indicating it produces proteases (33–35).

The Fusobacteriaceae were represented by phylotype PR6, which responded late in the protein treatment and had a 96% sequence identity to its closest cultured relative Cetobacterium somerae (Fig. 5B). Although a 96% sequence identity is relatively low in terms of species-level classification, it is of interest to note that C. somerae cannot hydrolyze complex proteins itself but is able to ferment amino acids and peptides to acetate, propionate, and butyrate and occurs in gastrointestinal systems (37, 38). Fusobacteriaceae-affiliated sequences with identities of up to 99% to phylotype PR6 (HG964632) (Fig. 5B) were also present in the gut contents of the epigeic earthworm Eudrilus eugeniae (11). This finding and the protein-linked response of Fusobacteriaceae-affiliated phylotype PR6 in the gut contents of anecic L. terrestris indicates that this family may contribute to the degradation of amino acids in earthworms of contrasting feeding guilds.

Phylotypes that displayed a more moderate response to protein were most closely related to proteolytic anaerobes, including Clostridium thiosulfatireducens (phylotype PR7), Clostridium difficile (phylotype PR8), and Clostridium tunisiense (phylotype PR12) (39–41). Acetate, methyl butyrate, propionate, and butyrate are common products of amino acid fermentations (24) and were formed in protein treatments. Furthermore, the phylotypes detected in these treatments were closely related to species that fermentatively produce these fatty acids (33, 35, 37, 38, 42–44).

Thus, many phylotypes that responded to protein were affiliated with proteolytic taxa. In this regard, although protein can be provided by the gizzard disruption of cells, protein is also a component of the gut mucus (3, 45, 46), further evidence that protein is available in the alimentary canal and therefore subject to utilization during gut passage.

Phylotype PR3, the dominant phylotype that responded to RNA (Fig. 5B), was closely affiliated with Aeromonas (100% identity to Aeromonas *media* and Aeromonas hydrophila), a genus present in gut contents of L. terrestris (13, 47) and casts of Lumbricus rubellus (48). Ribose, the RNA-associated pentose, stimulated fermentation. Aeromonas-affiliated facultative aerobes can hydrolyze RNA and convert pentoses to acetate, succinate, and formate (26, 49–52), products that were formed in the RNA and ribose treatments (Fig. 3; see also Table S7 in the supplemental material). Likewise, Aeromonadaceae-affiliated taxa in gut contents of L. terrestris have the ability to ferment the pentose xylose (13). These findings reinforce the likelihood that ribose was important to the observed response of this family to RNA (Fig. 4). It is probable that RNases are produced by ingested soil microbes in response to RNA, since soil microbes have been shown to produce extracellular RNases (53, 54).

On the basis of the increase in relative abundance of 16S rRNA sequences, the Enterobacteriaceae and Lachnospiraceae responded to cell lysate but appeared to be nonresponsive to protein or RNA (Fig. 5A), suggesting that nutrients other than protein and RNA in cell lysate stimulated additional taxa and associated processes not linked to either of these biopolymers. Cell lysate contains many components in addition to protein and RNA, including diverse saccharides (16, 18). In this regard, the alimentary canal of L. terrestris contains Enterobacteriaceae-affiliated fermenters that can ferment gut-associated saccharides (13), suggesting that the strong response of Enterobacteriaceae-affiliated phylotype CL4 to cell lysate (Fig. 5B) might have been due to lysate-derived saccharides. Indeed, the closely related phylotype PR33 did not respond to protein but displayed a modest response to RNA, a finding consistent with saccharides (ribose from RNA) being potentially utilized by these Enterobacteriaceae-affiliated phylotypes. Clostridia are classic consumers of saccharides, and several clostridial phylotypes responded only to the lysate treatment. These phylotypes included CL5 and CL18, which were most closely related to the acetogens Clostridium glycolicum and Clostridium magnum, respectively (55, 56). Acetogens occur in the gut contents of the methane-emitting earthworm E. eugeniae (11). Hydrogenotrophic methanogens also occur in E. eugeniae (11), but the gut contents of L. terrestris do not display any methanogenic potential (13). Although the apparent consumption of formate might have been associated with acetogenesis, nonacetogenic formate-hydrogen-lyase-containing taxa might have also been associated with formate consumption (e.g., Enterobacteriaceae-affiliated phylotypes PR33 and CL4 [57]). On the assumption that acetogens were active in the lysate treatment, the large continual production of H_2_ suggests that the amount of H_2_ formed by various fermentations exceeded the H_2_-consumming capacity of acetogens.

Phylotype CL2 (99% identity to the amino acid and carbohydrate fermenter Clostridium bifermentans [33]) responded rapidly to cell lysate during the first 6 h of incubation but subsequently decreased in relative abundance, whereas phylotypes CL8 (99% identity to the proteolytic fermenter Clostridium peptidivorans [30]) and CL6 (95% identity to the potentially proteolytic Lachnospiraceae-affiliated fermenter Niameybacter massiliensis [58]) had more sustained responses to cell lysate, yielding maximum relative abundances of 16S rRNA at the end of the 30-h incubation (Fig. 5B). This pattern might reflect the capacity of fermenters with broad substrate spectra to initially be more competitive for the diversity of substrates available from cell lysate. The responses of the closely related phylotypes CL2 and PR2 in controls lacking supplements were different, with the response of CL2 being more pronounced, a finding that might be due in part to a difference in the nutrient status of the gut contents at the time of gut content harvest.

### Conclusions, limitations, and future perspectives.

**FIG 6:**
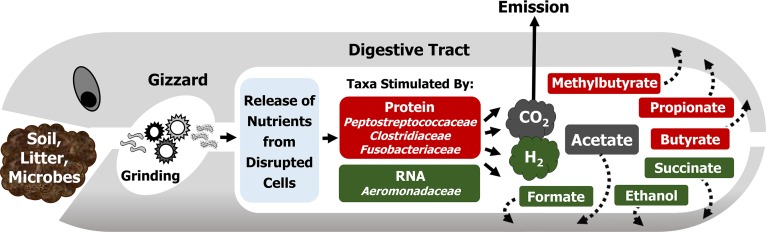
Hypothetical model illustrating the potential trophic interactions between the earthworm L. terrestris and ingested soil microorganisms capable of fermenting protein- and RNA-derived organic carbon, a source of which can be gizzard-disrupted cells. Broken arrows symbolize the utilization of fermentation products by the earthworm.

The collective findings indicate that protein and RNA, primary components of disrupted microbial cells, can stimulate subsets of gut content-associated fermentative taxa, and Fig. 6 illustrates the potential trophic interactions between the earthworm and such taxa in the alimentary canal. The model is a theoretical abstraction of the main findings and does not depict all anaerobic processes in the alimentary canal (e.g., denitrification and polysaccharide-linked fermentation are not shown [8, 9]). As such, the model emphasizes that protein and RNA may contribute to the overall fermentation profile of the alimentary canal of the earthworm. The relatively short read length obtained by Illumina sequencing can compromise accurate species-level taxonomic classification (59, 60), and the model has therefore been restricted to family-level identities of the main taxa that responded in the fermentation of protein and RNA. Protein-based fermentation occurs in other gut ecosystems. For example, the fermentation of protein in the gastrointestinal tract of higher animals, including humans, can affect the functional status of gut microbiota and the health status of the animal (61–63). We are not aware of another study that has evaluated microbial taxa that facilitate RNA-based fermentation in a gut ecosystem.

The experimental design did not simulate all of the *in situ* conditions of the gut, and the quantitative differences observed for the contrasting phylotypes cannot be extended to *in vivo* conditions. As such, the model does not exclude the possibility that less responsive taxa also participated in the protein- and RNA-based fermentation, and likewise does not address what taxa might respond to low nutrient input. However, the findings qualitatively illustrate the potential competiveness of subsets of the fermentative taxa that could respond to protein- and RNA-derived organic carbon and thus contribute to gut-associated fermentations. In this regard, the rapid stimulation of phylotypes CL2, CL7, PR2, and PR3 (Fig. 5B) illustrate the marked anaerobic abilities of phylotypes that are related to bacteria with phenotypes that are consistent with the fermentation profiles obtained. The proposed emission of fermentation-derived H_2_ is consistent with the occurrence of H_2_ in the gut and concomitant *in vivo* emission of H_2_ by L. terrestris (9), an activity potentially linked to secondary H_2_-consuming processes in soil (64, 65). The proposed emission of fermentation-derived CO_2_ is less certain and would in part be influenced by the formation of carbonates in the alimentary canal and worm tissues.

The maximum recorded densities of earthworms in soil theoretically yield up to 500 ml gut content per square meter of soil (11, 66), and the alimentary canal can be conceptualized as an anoxic microzone through which ingested soil microbes pass (5). How the earthworm gut influences the turnover of biopolymers at the microbiological level in the terrestrial biosphere is largely unresolved, but the present study indicates that this anoxic microzone can facilitate protein- and RNA-based fermentations. These microbial fermentations yield fatty acids that could be subsequently utilized by the earthworm (Fig. 6) (14). However, the earthworm would also benefit from assimilating the initial products of biopolymer hydrolysis (e.g., protein-derived amino acids) prior to microbial fermentation. Thus, there is likely an *in situ* competition between the earthworm and gut fermenters for the initial products of biopolymer hydrolysis. In this regard, earthworm salivary glands secrete proteases into the alimentary canal, indicating that the earthworm contributes to the breakdown of protein during gut passage (17). As such, certain nonproteolytic amino acid fermenters likely benefit from the protease-rich gut.

Despite the 10-fold dilution of gut content needed to facilitate the sampling of the aqueous phase of the anoxic microcosms, the protein-, RNA-, and cell lysate-dependent stimulations of both 16S rRNA synthesis and fermentation were rapid (i.e., occurred within the initial 6 to 10 h of incubation), demonstrating that such substrates have the potential to stimulate microbes during gut passage, which varies from 8 to 24 h depending on the earthworm species and its feeding status (47, 67, 68). DNA can constitute up to 3% of the dry weight of microbial cells (16, 18) and is therefore another nucleic acid released from gizzard-disrupted cells. Given the marked potential of RNA to stimulate fermentation, it seems likely that the hydrolysis of DNA could also contribute to fermentation in the gut. Current studies are focused on resolving which gut-associated fermentative taxa might be capable of utilizing other biopolymers and breakdown products thereof and how these processes might contribute to the gut microbiology of the earthworm.

## MATERIALS AND METHODS

### Earthworms.

L. terrestris specimens were purchased from ANZO (Bayreuth, Germany) and maintained in loamy soil from the meadow Trafo-Wiese in Bayreuth (12). Earthworms were kept on soil at 20°C for approximately 10 days prior to use; fresh grass and foliage served as feedstock.

### Cell-free lysate.

S. cerevisiae strain Sa-07140 (DSMZ, Braunschweig, Germany) was cultivated at 30°C in autoclaved oxic medium (pH 7) containing per liter: 7 g yeast extract, 7 g tryptic soy broth, and 10 g glucose. E. coli K-12 (DSMZ, Braunschweig, Germany) was cultivated at 37°C in autoclaved oxic medium (pH 7) containing per liter: 8 g tryptone, 8 g yeast extract, 5 g sodium chloride, and 5 g glucose. Cells were harvested in late exponential phase by centrifugation for 20 min at 7,500 rpm (approximately 10,000 × *g* [J2-HS-centrifuge, JA10-rotor; Beckmann, Fullerton, CA, USA]). The cell pellets were washed three times with sodium phosphate buffer (36 mM, pH 7). Twenty grams fresh weight of pelleted cells was suspended in 20 ml sodium phosphate buffer, and 400 μl DNase I (10,000 U · ml^−1^ [Sigma]) was added. The cell suspension was subjected to three consecutive runs through a French press (95,000 to 110,000 kPa [FA-032-40K pressure cell; SLM Aminco, Urbana, IL, USA]). The ruptured cells were centrifuged for 20 min at 15,000 rpm (approximately 27,000 × *g* [J2-HS-centrifuge, JA20-rotor; Beckmann]) and the pellet was discarded. The supernatant fluid was centrifuged again. Approximately 27 ml of the supernatant fluid was diluted with 13 ml sodium phosphate buffer, filter sterilized (0.2 μm pore size, cellulose-acetate membrane [Sartorius Stedim, Göttingen, Germany]), and transferred to sterile anoxic 100-ml serum vials that were crimp sealed with sterile rubber stoppers (Glasgerätebau Ochs Laborfachhandel e.K., Bovenden, Germany); the vials were then flushed with sterile argon (100%). The dry weight of the cell lysate was determined by weighing before and after drying at 60°C for 7 days (12, 47). The amount of carbon per milliliter cell lysate was calculated on the basis of the dry weight and molar masses of (i) 26.2 g · mol^−1^ for S. cerevisiae biomass (according to the chemical formula [CH_1.613_O_0.557_N_0.158_P_0.012_S_0.003_K_0.022_Mg_0.003_Ca_0.001_]_n_) and (ii) 24.2 g · mol^−1^ for E. coli biomass (according to the chemical formula [CH_1.59_O_0.374_N_0.263_P_0.0234_S_0.006_]_n_) (69).

### Protein and RNA stock solutions.

The following theoretical chemical formulas for protein and RNA were used to calculate the amount of carbon provided in a given treatment: [CH_1.57_N_0.27_O_0.30_S_0.013_]_n_ for protein (i.e., bovine serum albumin) and [C_9.5_H_11.75_N_3.75_O_7_P]_n_ for RNA (based on 50% GC content and deprotonated phosphate). The 10-fold concentrated stock solutions of protein (22.5 mg/ml; bovine serum albumin [Merck, Darmstadt, Germany]) were prepared by adding the respective amounts to 10 ml autoclaved anoxic sodium phosphate buffer. RNA (from the yeast Cyberlindnera jadinii; Sigma) was less soluble than protein, and less concentrated stock solutions (8.8 mg/ml) were prepared, and the pH was adjusted to pH 7 with 1 M NaOH. Stock solutions of protein and RNA were filter sterilized (0.2 μm pore size, cellulose-acetate membrane) and transferred to sterile anoxic 100-ml serum vials that were crimp sealed with sterile rubber stoppers; the vials were then flushed with sterile argon (100%). According to the manufacturer's specifications, the RNA contained ≤10% water, which was neglected for all calculations.

### Anoxic microcosms.

Worms were anesthetized on ice with CO_2_, and gut content microcosms were prepared in an O_2_-free chamber (100% N_2_ gas phase [Mecaplex, Grenchen, Switzerland]) as described previously (47). Microcosms were prepared in 27-ml sterile glass tubes. One gram fresh weight of gut content (pooled from 25 to 30 individuals) was supplemented with anoxic fresh cell lysate (1 ml) or anoxic stock solutions of protein (1 ml) or RNA (3.83 ml), and anoxic sodium phosphate buffer was added to a total volume of 10 ml for each microcosm. Control treatments contained gut content and sodium phosphate buffer but no substrate. The tubes were closed with sterile rubber stoppers, crimp sealed, and then flushed and pressurized (60 kPa) with sterile N_2_. Three replicate microcosms per treatment were incubated in the dark at room temperature (approximately 21 to 24°C). Sampling was with sterile syringes.

### Chemical analyses.

A WTW pH 323 pH meter (Zeller, Hohenems, Austria) was used to measure pH. Gases and dissolved organic compounds were measured by gas chromatography and high-performance liquid chromatography, respectively; additional information on the instrumentation is provided in Table S9 in the supplemental material (70). Amounts of H_2_ and CO_2_ in the gas and liquid phases were calculated from the ideal gas law and standard solubility tables (71); for CO_2_, the amounts of bicarbonate (calculated from dissolved CO_2_, pH, and the dissociation constant) were taken into consideration. The final amounts of gases and organic compounds were normalized to the fresh weight of gut content. One micromole of a compound per gram of fresh weight corresponds to 0.1 mmol per liter of microcosm.

### Theoretical recoveries of carbon and reducing equivalents.

For recoveries derived from cell lysate, the amounts of gases or organic compounds formed in unsupplemented controls were subtracted from those of biopolymer or cell lysate treatments to obtain net amounts of a certain compound X (n_net_X). n_net_X was multiplied by the number of carbon atoms and the number of reducing equivalents (i.e., the number of electrons obtained by complete oxidation of compound X) to calculate the amount of carbon (n_c_X) and the amount of reducing equivalents (n_r_X) recovered in compound X, respectively (numbers of carbon atoms/reducing equivalents were: acetate, 2/8; butyrate, 4/20; CO_2_, 1/0; formate, 1/2; hydrogen, 0/2; methyl butyrate, 5/26; propionate, 3/14; succinate, 4/14; lactate, 3/12; ethanol, 2/12). n_c_X was divided by the total amount of carbon supplemented as the substrate (n_c_S) to obtain final carbon recoveries. The final recoveries of reducing equivalents were calculated by dividing n_r_X by the total amount of reducing equivalents supplemented as the substrate (n_r_S). n_r_S was obtained by multiplying n_c_S by the number of theoretical reducing equivalents per carbon atom of the substrate (n_r_C). n_r_C was 4.019 for cell lysate (based on the chemical formula [CH_1.613_O_0.557_N_0.158_]_n_ for S. cerevisiae biomass), yielding an average oxidation level of −0.019 for carbon (69). For recoveries derived from protein and RNA, n_r_C was (i) 4.145 for protein (based on the chemical formula [CH_1.57_N_0.27_O_0.30_S_0.013_]_n_ for bovine serum albumin, yielding an average oxidation level of −0.145 for carbon) and (ii) 3.1 for RNA (based on the chemical formula [C_9.5_H_11.75_N_3.75_O_7_]_n_ for RNA, yielding an average oxidation level of +0.9 for carbon).

### DNA/RNA extraction.

The solid phase of microcosms was obtained by centrifugation (5 min at 4°C, 15,000 × *g* [1-15-K Sartorius]). RNA and DNA were coextracted from 0.2 to 0.8 g fresh weight of pelleted material (from either pooled or individual replicates) by bead-beating lysis (Fast Prep FP120, BIO101 Thermo Savant, Carlsbad, CA, USA), organic solvent extraction, and precipitation (72). Enzymatic digestions with RNase A (30 min at room temperature, 10 μg · μl^−1^ [Fermentas, St. Leon-Roth, Germany]) or DNase I (45 min at 37°C, 1 U · μl^−1^ [Fermentas]) yielded DNA or RNA, respectively. No 16S RNA gene-specific PCR products were obtained from RNA samples, indicating that DNA digestion was successful.

### Reverse transcription-PCR.

RNA was transcribed into cDNA using random hexamers (Microsynth, Balgach, Switzerland) and SuperScript III reverse transcriptase (cDNA synthesis kit; Invitrogen) according to the manufacturer's protocol. Isopropanol precipitation was performed to prepare DNA and cDNA samples for storage and shipment (73).

### Sequence analyses.

DNA and cDNA were sent to Microsynth for PCR amplification, amplicon sequencing, and initial sequence analyses. Amplifications of the V3 and V4 regions of 16S rRNA genes were with primers Bakt_341F (5′-CCTACGGGNGGCWGCAG-3′) and Bakt_805R (5′-GACTACHVGGGTATCTAATCC-3′), yielding PCR products of approximately 460 bp that covered most bacterial linages (74). The amplified region is considered suitable for bacterial community analyses (75). Due to mismatches of primers Bakt_341F and Bakt_805R to the mitochondrial 16S rRNA genes of Saccharomyces cerevisiae (i.e., the source of the cell lysate) or Cyberlindnera jadinii (i.e., the source of the purchased RNA), an undesired amplification of substrate-derived 16S rRNA sequences was not possible and was also not observed.

The following protocol was used for PCR amplification: initial denaturation, 3 min at 95°C; 20 cycles of denaturation (20 s, 98°C), annealing (30 s, 56°C), and elongation (30 s, 72°C); final elongation, 5 min at 72°C. A Kapa HiFi HotStart PCR kit (Kapa Biosystems, Wilmington, MA, USA) was used per the manufacturer's protocol.

Amplicon sequencing was by Illumina MiSeq. Raw sequences were demultiplexed, stitched, quality filtered, and checked for chimeras using the QIIME software package (76). For experiments with supplemental cell lysate, nonchimeric sequences were clustered into phylotypes on the basis of a sequence similarity cutoff of 99%. To reduce the number of phylotypes that potentially result from erroneous Illumina sequencing (77), a more conservative similarity cutoff of 97% was applied for the clustering of nonchimeric sequences in experiments with supplemental protein or RNA. The resulting phylotypes were classified with the SILVA incremental aligner SINA (78, 79). Singeltons were excluded.

Representative sequences of phylotypes with ≥0.1% relative abundance were reanalyzed with the ARB software package (80). Highly similar phylotypes (≥97% nucleic acid similarities) were identified by calculating a similarity matrix and were merged. Representative sequences of responsive phylotypes and closely related reference sequences were used to calculate phylogenetic trees (phylotypes were designated responsive when a phylotype in a given treatment displayed a minimum increase in relative abundance of 2% above the control values in at least one of the sampling periods).

### Sequence abundances.

The relative abundances of all sequences, including less abundant sequences not highlighted in Results, are provided in Table S10 (lysate treatment) and Table S11 (protein and RNA treatments).

### Accession number(s).

The sequences were deposited at the European Nucleotide Archive (ENA) under study numbers PRJEB15377 and PRJEB15410 for cell lysate and protein/RNA treatments, respectively. Representative sequences of phylotypes with ≥0.1% relative abundance were deposited under accession numbers LT626667 to LT626823 (cell lysate treatment) and LT626824 to LT626940 (protein/RNA treatments).

## Supplementary Material

Supplemental material
