# Supplementary material for: Protein- and RNA-Enhanced Fermentation by Gut Microbiota of the Earthworm Lumbricus terrestris
Source: Appl Environ Microbiol. 2018 May 17;84(11):e00657-18. doi: 10.1128/AEM.00657-18 (PMC5960956; doi:10.1128/AEM.00657-18)
Supplement: Supplemental material [file supp_84_11_e00657-18__index.html]

Supplemental material 

# Protein- and RNA-Enhanced Fermentation by Gut Microbiota of the Earthworm Lumbricus terrestris

## Supplemental material

- Supplemental file 1 -

  Rarefaction analyses of bacterial 16S rRNA and 16S rRNA gene sequences obtained from cell-lysate and protein and RNA treatments (Fig. S1); effect of biopolymers on the formation of H2 and CO2 in anoxic microcosms of *Lumbricus terrestris* gut contents (Fig. S2); effect of cell lysates from *Saccharomyces cerevisiae* and *Escherichia coli* on the formation of CO2 and H2 in anoxic microcosms of *L. terrestris* gut contents (Table S1); statistical *P* values of the products formed in *S. cerevisiae* lysate, protein, and RNA treatments (Table S2); time-resolved alpha diversity of the microbial community in the *S. cerevisiae* lysate treatment (Table S3); statistical *P* values of abundant families that were stimulated by supplemental lysate, protein, or RNA (Table S4); fatty acid profiles of anoxic microcosms of *L. terrestris* gut contents supplemented with different biopolymers (Table S5); effect of different amounts of protein or RNA on the formation of CO2 or H2, respectively, in anoxic microcosms of *L. terrestris* gut contents (Table S6); fermentation profiles and estimated recoveries of anoxic microcosms of *L. terrestris* gut contents supplemented with Casamino acids or ribose (Table S7); time-resolved alpha diversity of the microbial community in protein and RNA treatments (Table S8); instrumentation utilized for analyses of organic acids and gases (Table S9); summary of families of the *S. cerevisiae* lysate treatment (Table S10); summary of families of the protein and RNA treatments (Table S11).

  PDF, 171K
