# Supplementary material for: Protein- and RNA-Enhanced Fermentation by Gut Microbiota of the Earthworm Lumbricus terrestris
Source: Appl Environ Microbiol. 2018 May 17;84(11):e00657-18. doi: 10.1128/AEM.00657-18 (PMC5960956; doi:10.1128/AEM.00657-18)
Supplement: Supplemental material [file AEM.00657-18_zam011188533s1.pdf]

Supplemental Material

Protein- and RNA-Enhanced Fermentation by Gut Microbiota of the Earthworm *Lumbricus terrestris*

Lydia Zeibich, Oliver Schmidt, and Harold L. Drake*

Department of Ecological Microbiology, University of Bayreuth, 95440 Bayreuth, Germany

*Corresponding author: Harold L. Drake, Department of Ecological Microbiology, University of Bayreuth, 95440 Bayreuth, Germany. Tel: (+49) (0)921-555640. Fax: (+49) (0)921-555793. E-Mail: HLD@Uni-Bayreuth.De

List of Supplemental Figures and Tables

Supplemental Figures

Figure S1. Rarefaction analyses of bacterial 16S rRNA and 16S rRNA gene sequences obtained from (A) cell-lysate and (B) protein and RNA treatments.

Figure S2. Effect of biopolymers on the formation of H_2 and CO_2 in anoxic microcosms of *L. terrestris* gut contents.

Supplemental Tables

Table S1. Effect of cell lysates from *S. cerevisiae* and *E. coli* on the formation of CO_2 and H_2 in anoxic microcosms of *L. terrestris* gut contents.

Table S2. Statistical *P* values of the products formed in (A) *S. cerevisiae* lysate, (B) protein, and (C) RNA treatments.

Table S3. Time-resolved alpha diversity of the microbial community in the *S. cerevisiae* lysate treatment.

Table S4. Statistical *P* values of abundant families that were stimulated by supplemental lysate, protein, or RNA.

Table S5. Fatty acid profiles of anoxic microcosms of *L. terrestris* gut contents supplemented with different biopolymers.

Table S6. Effect of different amounts of protein or RNA on the formation of CO_2 or H_2, respectively, in anoxic microcosms of *L. terrestris* gut contents.

Table S7. Fermentation profiles (A) and estimated recoveries (B) of anoxic microcosms of *L. terrestris* gut contents supplemented with casamino acids or ribose.

Table S8. Time-resolved alpha diversity of the microbial community in protein and RNA treatments.

Table S9. Instrumentation utilized for analyses of organic acids and gases.

Table S10. Summary of families of the *S. cerevisiae* lysate treatment (the most abundant taxa are displayed in Figure 2).

Table S11. Summary of families of the protein and RNA treatments (the most abundant taxa are displayed in Figure 4).

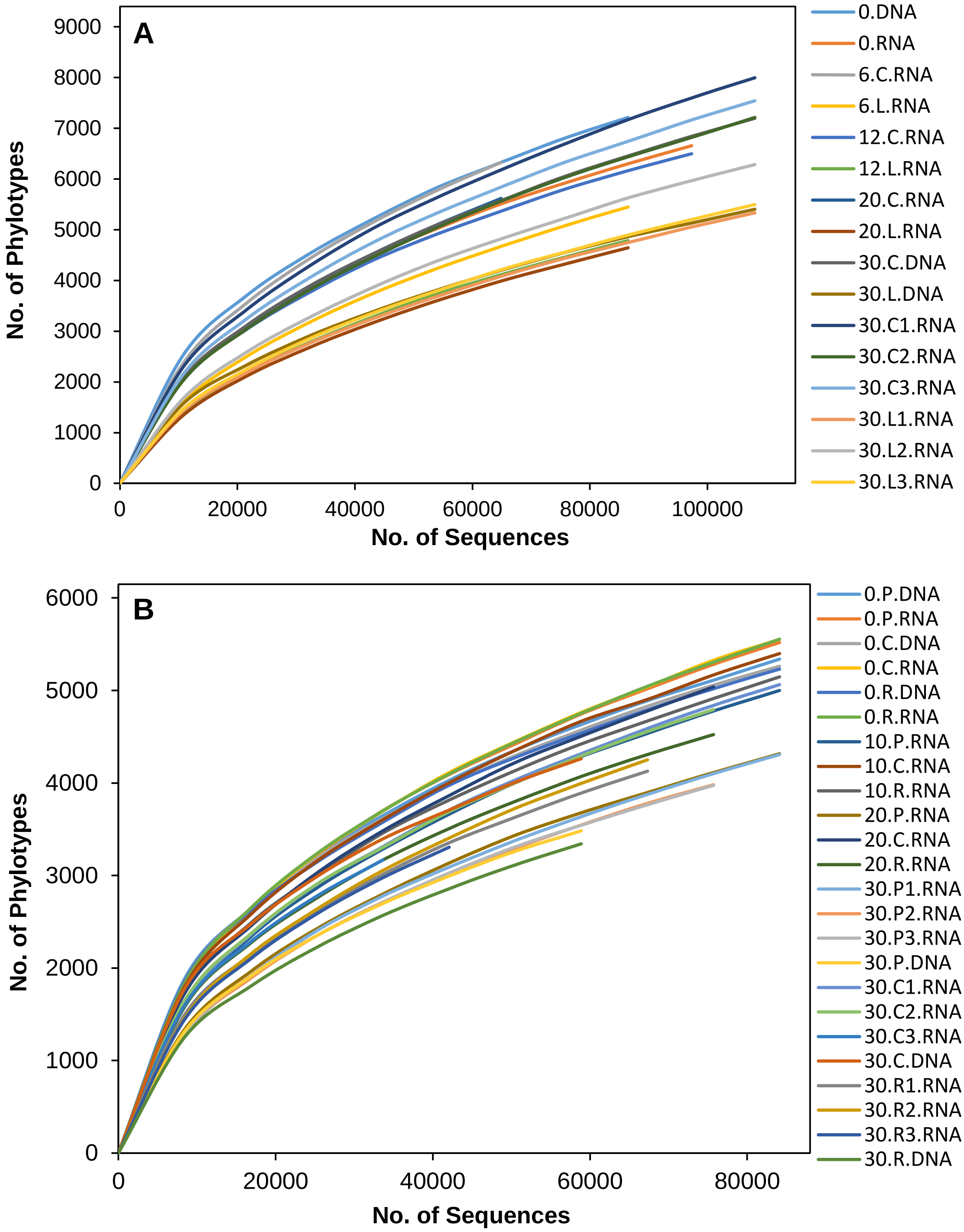

Figure S1. Rarefaction analyses of bacterial 16S rRNA and 16S rRNA gene sequences obtained from (A) cell-lysate treatment and (B) protein and RNA treatments. Phylotypes were based on a 99% (panel A) and 97% (panel B) sequence similarity cutoff. Abbreviations: 0, 6, 10, 12, 20, 30 indicate the time of sampling in hours; C, unsupplemented control; L, lysate treatment; P, protein treatment; R, RNA treatment. For both panels, RNA or DNA samples of the three replicates were always pooled except for RNA samples at 30 hour, the identification numbers (e.g., C1) of which indicate the respective replicates.

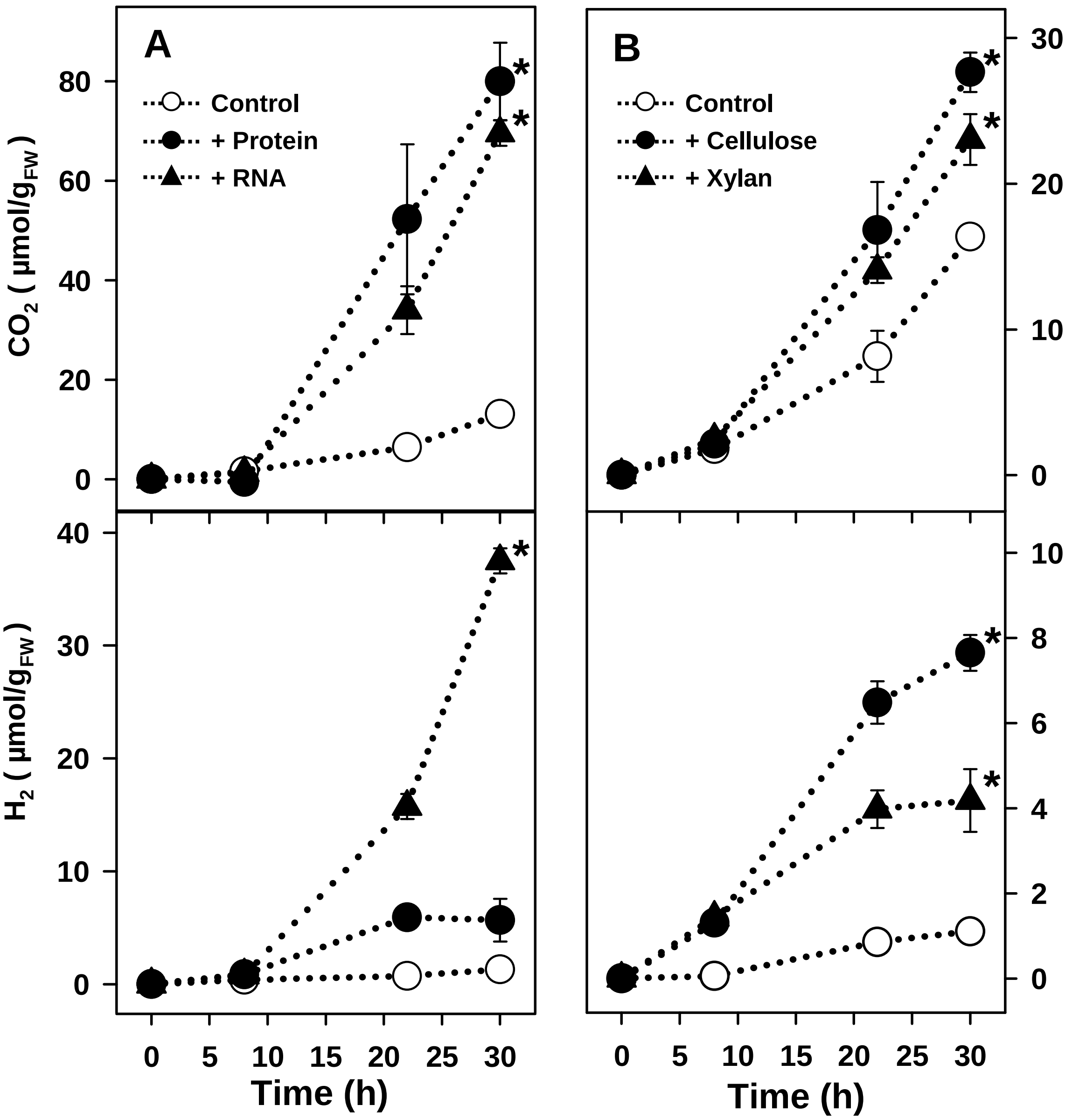

Figure S2. Effect of biopolymers on the formation of H_2 and CO_2 in anoxic microcosms of *L. terrestris* gut contents. The amount of biopolymer-derived carbon added per microcosm (10 ml containing 1 g_{FW} gut content) approximated 2.4 mmoles (corresponds to 54 mg protein, 81 mg RNA, 68 mg cellulose, or 63 mg xylan). Controls lacked supplemental biopolymers. Values are the arithmetic average of three replicate analyses, and error bars indicate the standard deviations. Some standard deviations are smaller than the size of the symbol and therefore not apparent. The asterisk (*) indicates significant *P* values ($P \leq 0.05$) of control vs. protein, RNA, cellulose, or xylan treatments at the end of incubation. *P* values were calculated by t-Test with unequal variances. FW, fresh weight.

Table S1. Effect of cell lysates from *S. cerevisiae* and *E. coli* on the formation of CO_2 and H_2 in anoxic microcosms of *L. terrestris* gut contents.

Treatment[a]		pH		Gaseous products (µmol/g_{FW})			
				**CO_2**		**H_2**	
	Replicate	1	2	1	2	1	2
Control		6.8	6.9	18.6	21.6	2.2	2.4
E. coli		6.6	6.6	60.4	55.3	5.9	9.7
S. cerevisiae		6.6	6.6	63.8	63.6	14.6	15.7

[a]The amount of carbon derived from filter sterilized *S. cerevisiae* lysate (5.1% dry weight) and *E. coli* lysate (5.3% dry weight) added per microcosm (10 ml containing 1 g_{FW} gut content) approximated 2.0 mmoles and 2.2 mmoles, respectively. Filter-sterilized lysate alone did not display any fermentation activity. Control lacked supplemental lysate. Earthworms from which gut contents were obtained were maintained at 15℃, and microcosms were incubated at 15℃ for 44 hours. FW , fresh weight.

Table S2. Statistical *P* values of the products formed in (A) *S. cerevisiae* lysate, (B) protein, and (C) RNA treatments.

(A) Lysate treatment (Figure 1)[a]

	Products																	
	CO_2		H_2		Succinate		Lactate		Formate		Acetate		Propionate		Butyrate		Methylbutyrate	
	C	L	C	L	C	L	C	L	C	L	C	L	C	L	C	L	C	L
**Mean value[b]**	30	149	0.9	33	4.1	58	1.3	1.5	6.2	48	29	262	7.7	14	2.0	13	10	96
**Variance**	479	1046	0.0	3.8	0.0	142	0.0	0.0	0.9	50	31	2656	3.2	0.2	0.1	0.1	1.6	88
***P* value[c]**	0.001		0.006		0.016		0.103		0.009		0.016		0.030		0.000		0.004	

(B) Protein treatment (Figure 3)[a]

	CO_2		H_2		Succinate		Lactate		Formate		Acetate		Propionate		Butyrate		Methylbutyrate	
	C	P	C	P	C	P	C	P	C	P	C	P	C	P	C	P	C	P
**Mean value[b]**	7.1	38	0.5	3.2	0.0	0.0	0.3	0.4	1.5	11	15	90	3.5	19	0.9	11	3.0	30
**Variance**	0.2	3.5	0.0	0.1	0.0	0.0	0.0	0.0	0.1	0.7	0.2	7.4	0.1	0.5	0.0	0.0	0.0	8.6
***P* value[c]**	0.001		0.001		-		0.502		0.016		0.010		0.000		0.009		0.004	

(C) RNA treatment (Figure 3)[a]

	CO_2		H_2		Succinate		Lactate		Formate		Acetate		Propionate		Butyrate		Methylbutyrate	
	C	R	C	R	C	R	C	R	C	R	C	R	C	R	C	R	C	R
**Mean value[b]**	7.1	76	0.5	48	0.0	13	0.3	6.1	2.8	26	15	59	3.5	3.7	0.9	0.4	3.0	2.1
**Variance**	0.2	39	0.0	9.6	0.0	0.1	0.0	0.0	0.1	0.4	0.2	4.8	0.1	0.0	0.0	0.0	0.0	0.4
***P* value[c]**	0.001		0.003		0.000		0.000		0.000		0.001		0.775		0.118		0.158	

[a]C, L, P, and R corresponds to unsupplemented control, lysate, protein, and RNA treatments.

[b]Mean value (n = 3) of the different fermentation products in µmol/g_{FW}. FW, fresh weight.

[c]*P* values were calculated by t-Test with unequal variances. Values are significant at $P \leq 0.05$.

Table S3. Time-resolved alpha diversity of the microbial community in the *S. cerevisiae* lysate treatment.

**Sampling time:**	**0 h**		**6 h**		**12 h**		**20 h**	
**Treatment:**[a]	**DNA**	**RNA**	**C.RNA**	**L.RNA**	**C.RNA**	**L.RNA**	**C.RNA**	**L.RNA**
Number of sequences	90591	105034	70681	90615	104319	87844	70036	96059
Observed PTs[b] (normalized)[c]	7384 (6328)	6911 (5506)	6625 (6326)	5596 (4670)	6720 (5365)	4833 (4112)	5848 (5618)	4917 (3989)
Chao1 (normalized)[c]	11975 (10608)	11553 (9751)	11842 (11419)	10159 (8995)	11199 (9460)	8722 (7864)	10712 (10409)	9341 (7911)
Shannon (normalized)[c]	9.8 (9.7)	7.3 (7.3)	8.2 (8.1)	6.1 (6.0)	7.9 (7.9)	5.8 (5.8)	7.2 (7.2)	6.2 (6.2)

**Sampling time:**	**30 h**							
**Treatment:**[a]	**C1.RNA**	**C2.RNA**	**C3.RNA**	**L1.RNA**	**L2.RNA**	**L3.RNA**	**C.DNA**	**L.DNA**
Number of sequences	109807	147651	126676	114515	126281	142222	111359	122114
Observed PTs[b] (normalized)[c]	8086 (6179)	8411 (5566)	8150 (5841)	5509 (4083)	6825 (4822)	6332 (4214)	7323 (5573)	5745 (4200)
Chao1 (normalized)[c]	14324 (11675)	14417 (10764)	13660 (10822)	10514 (8101)	12345 (9390)	11492 (8483)	12972 (10395)	9945 (7761)
Shannon (normalized)[c]	8.3 (8.3)	7.6 (7.6)	7.7 (7.6)	6.6 (6.6)	7.2 (7.1)	6.7 (6.7)	8.3 (8.2)	7.2 (7.2)

[a]C and L corresponds to unsupplemented control and cell lysate treatments, respectively. RNA or DNA samples of the three replicates were always polled except for RNA samples at 30 h (the numbers of each treatment at 30 h [e.g., C1] indicate the respective replicate). DNA, 16S rRNA genes; RNA, 16S rRNA.

[b]Phylotypes (PTs) were clustered based on a sequence similarity cut-off of 99%.

[c]The data sets were normalized to 64,864 sequences for comparison of amplicon libraries of different sizes.

Table S4. Statistical *P* values of abundant families that were stimulated by supplemental lysate, protein, or RNA.[a]

(A) Lysate Treatment (Figure 2)

Family	Treatment	Mean	Variance	*P* value[b]
Aeromonadaceae	Control	0.2	0.0	0.010
	Lysate	1.4	0.0	
Clostridiaceae	Control	1.3	0.0	0.007
	Lysate	11.9	2.4	
Enterobacteriaceae	Control	0.3	0.0	0.011
	Lysate	12.6	4.9	
Lachnospiraceae	Control	0.4	0.0	0.018
	Lysate	9.7	4.8	
Peptostreptococcaceae	Control	10.3	5.6	0.003
	Lysate	23.1	6.1	

(B) Protein and RNA Treatments (Figure 4)

Family	Treatment	Mean	Variance	*P* Value[b]
Aeromonadaceae	Control	0.2	0.0	
	RNA	19.1	5.5	0.005
	Protein	0.4	0.0	0.119 (0.005)
Clostridiaceae	Control	1.5	0.2	
	Protein	12.9	1.3	0.000
	RNA	3.3	0.2	0.009 (0.001)
Fusobacteriaceae	Control	1.0	0.3	
	Protein	8.4	4.7	0.029
	RNA	0.2	0.0	0.12 (0.023)
Peptostreptococcaceae	Control	4.6	0.5	
	Protein	22.5	5.8	0.006
	RNA	2.9	0.4	0.035 (0.005)

[a]Families were designated (a) abundant when a family had a relative abundance of ≥ 5% in at least one sampling period and (b) stimulated when the increase in relative abundance over time was more pronounced in at least one treatment (lysate, protein, or RNA) compared to the respective unsupplemented control.

[b]*P* values of control vs. lysate, protein, or RNA treatments were calculated from relative abundances at the end of the 30 h incubation by t-Test with unequal variances (parenthetical values indicate *P* values of protein vs. RNA treatments). Values are significant at $P \leq 0.05$.

Table S5. Fatty acid profiles of anoxic microcosms of *L. terrestris* gut contents supplemented with different biopolymers.

Treatment[a]	Time (h)	Products (µmol/g_{FW})[b]						
		Acetate	Succinate	Formate	Propionate	Butyrate	Methyl-butyrate	Lactate
**(A) Protein and RNA**								
Control	0	2.2 ± 0.5	0.5 ± 0.0	3.6 ± 0.4	0.0 ± 0.0	0.0 ± 0.0	0.0 ± 0.0	0.6 ± 0.1
	30	23 ± 2.8	0.0 ± 0.0	2.9 ± 0.8	4.5 ± 0.3	1.9 ± 0.1	5.3 ± 0.1	0.5 ± 0.1
Protein	0	2.7 ± 0.1	0.4 ± 0.1	2.7 ± 0.3	0.0 ± 0.0	0.9 ± 0.2	0.0 ± 0.0	1.2 ± 0.2
	30	[illegible]46 ± 5.6*	0.0 ± 0.0	13 ± 9.2	22 ± 1.7*	25 ± 2.6*	53 ± 1.3*	5.8 ± 2.4
RNA	0	18 ± 1.4	4.9 ± 0.3	2.3 ± 0.7	0.0 ± 0.0	0.0 ± 0.0	0.0 ± 0.0	4.9 ± 0.3
	30	87 ± 5.3*	19 ± 1.4*	39 ± 1.3*	8.0 ± 0.9*	3.2 ± 1.4	5.5 ±1.2	12 ± 0.5*
**(B) Cellulose and Xylan**								
Control	0	1.2 ± 0.1	0.5 ± 0.1	3.2 ± 0.2	0.0 ± 0.0	0.0 ± 0.0	0.7 ± 0.3	0.3 ± 0.0
	30	25 ± 1.4	1.9 ± 0.2	2.5 ± 0.8	3.7 ± 0.1	0.6 ± 0.0	6.8 ± 0.2	0.8 ± 0.1
Cellulose	0	1.8 ± 0.2	0.4 ± 0.1	0.7 ± 0.3	0.0 ± 0.0	0.0 ± 0.0	0.0 ± 0.0	0.3 ± 0.1
	30	33 ± 7.0	3.1 ± 0.1*	2.8 ± 2.9	4.0 ± 0.2	0.7± 0.0	7.1 ± 0.3	2.0 ± 0.2*
Xylan	0	12 ± 0.7	0.6 ± 0.0	1.3 ± 0.4	0.0 ± 0.0	0.0 ± 0.0	0.0 ± 0.0	0.4 ± 0.0
	30	45 ± 4.4*	2.7 ± 0.1*	3.0 ± 0.1	4.3 ± 0.1*	0.9 ± 0.0*	7.2 ± 0.3	1.6 ± 0.1*

[a]The amount of biopolymer-derived carbon added per microcosm (10 ml containing 1 g_{FW} gut content) approximated 2.4 mmoles (corresponds to 54 mg protein, 81 mg RNA, 68 mg cellulose, or 63 mg xylan). Controls lacked supplemental biopolymers. Values are the arithmetic average of three replicate analyses (± standard derivation). Time resolved production of CO_2 and H_2 are shown in Supplemental Figure S2. FW, fresh weight.

[b]The asterisk (*) indicates significant *P* values ($P \leq 0.05$) of control vs. protein, RNA, cellulose, or xylan treatments at the end of incubation. *P* values were calculated by t-Test with unequal variances.

Table S6. Effect of different amounts of protein or RNA on the formation of CO_2 or H_2, respectively, in anoxic microcosms of *L. terrestris* gut contents.

Treatment	Carbon (mmol)[a]	µmol/g_{FW}[b]	*P* Value[c]
**Protein**		CO_2	
	0.0	10.3 ± 2.8	
	0.5	25.0 ± 2.0	0.002
	2.0	55.6 ± 5.1	0.000
**RNA**		H_2	
	0.0	0.8 ± 0.1	
	0.5	8.7 ± 1.8	0.002
	2.0	47.3 ± 9.3	0.001

[a]The amount of biopolymer-derived carbon added per 10 ml microcosm containing 1 g_{FW} gut content (one mmole carbon corresponds to 22.5 mg protein and 33.8 mg RNA).

[b]Amounts of CO_2 and H_2 at the end of incubation (30 h). Values are the arithmetic average of three replicate analyses (± standard deviation). FW, fresh weight.

[c]*P* values were calculated by t-Test with different variances and are based on the difference between the unsupplemented control and the supplemented treatment. Values are significant at $P \leq 0.05$.

Table S7. Fermentation profiles (A) and estimated recoveries (B) of anoxic microcosms of *L. terrestris* gut contents supplemented with casamino acids or ribose.

(A) Fermentation Profile[a]

Treatment	Time (h)	pH	Products (µmol/g_{FW})[b]									
			CO_2	H_2	Acetate	Ethanol	Succinate	Lactate	Formate	Propionate	Butyrate	Methyl-butyrate
**Control**	0	7.0 ± 0.0	0.0 ± 0.0	0.0 ± 0.0	2.4 ± 0.2	0.0 ± 0.0	0.7 ± 0.0	1.0 ± 0.1	1.0 ± 0.1	0.0 ± 0.0	0 .0 ± 0.0	0.0 ± 0.0
	30	7.0 ± 0.0	7.5 ± 1.0	0.7 ± 0.1	16 ± 0.4	0.0 ± 0.0	0.4 ± 0.1	0.8 ± 0.1	0.7 ± 0.1	3.0 ± 0.1	1 .3 ± 0.1	1.7 ± 0.1
**Casamino acids**[c]	0	7.0 ± 0.0	0.0 ± 0.0	0.0 ± 0.0	2.5 ± 0.1	0.0 ± 0.0	0.6 ± 0.0	1.3 ± 0.0	1.2 ± 0.2	0.0 ± 0.0	0 .0 ± 0.0	0.0 ± 0.0
	30	6.9 ± 0.0	33 ± 2.8*	3.8 ± 0.2*	90 ± 3.4*	0.0 ± 0.0	2.6 ± 0.7*	1.4 ± 0.3	3.2 ± 0.9*	16 ± 1.1*	8.7 ± 0.4*	14 ± 1.5*
**Ribose**[d]	0	7.0 ± 0.0	0.0 ± 0.0	0.0 ± 0.0	1.9 ± 0.5	0.0 ± 0.0	0.0 ± 0.0	0.9 ± 0.3	0.9 ± 0.2	0.0 ± 0.0	0 .0 ± 0.0	0.0 ± 0.0
	30	6.8 ± 0.0	12 ± 1.0*	3.9 ± 0.8*	32 ± 3.1*	19 ± 2.3*	1.9 ± 0.4*	1.3 ± 0.1*	7.8 ± 0.4*	3.1 ± 0.2	1.5 ± 0.2	2.0 ± 0.2

(B) Recoveries (%)[e]

		CO_2	H_2	Acetate	Ethanol	Succinate	Lactate	Formate	Propionate	Butyrate	Methyl-butyrate	Total
**Casamino acids**[c]	Carbon	2.1	na	12.3	-	0.8	0.0	0.2	3.2	2.5	5.0	**26.2**
	Reducing Equivalents	na	0.1	11.7	-	0.6	0.0	0.1	3.6	2.9	6.2	**25.3**
**Ribose**[d]	Carbon	3.5	na	24.0	26.9	2.3	2.0	5.2	0.1	0.6	1.2	**65.8**
	Reducing Equivalents	na	1.2	24.0	40.3	2.0	2.0	2.6	0.2	0.8	1.5	**74.6**

[a]Controls lacked supplemental substrates. Values are the arithmetic average of three replicate analyses (± standard derivation). FW, fresh weight.

[b]The asterisk (*) indicates significant *P* values ($P \leq 0.05$) of control vs. casamino acids or ribose treatments at the end of incubation. *P* were calculated by t-Test with unequal variances.

[c]The amount of casamino acid-derived carbon added per microcosm (10 ml containing 1 g_{FW} gut content) approximated 1.2 mmoles (corresponds to 25.2 mg casamino acids; based on the chemical formula of casein $[CH_{1.95}N_{0.25}O_{0.49}S_{0.007}]_n$, yielding an average oxidation level of -0.214 for carbon).

[d]The amount of ribose per microcosm approximated 5 mM, of which 3 mM were consumed.

[e]Recoveries were calculated at the end of the 30 hour incubation and are based on the arithmetic average of three replicate analyses. -, no net increase of the product during the 30 h incubation. na, not applicable.

Table S8. Time-resolved alpha diversity of the microbial community in protein and RNA treatments.

Sampling Time	0 h						10 h			20 h		
**Treatment:[a]**	**C.DNA**	**P.DNA**	**R.DNA**	**C.RNA**	**P.RNA**	**R.RNA**	**C.RNA**	**P.RNA**	**R.RNA**	**C.RNA**	**P.RNA**	**R.RNA**
Number of sequences	95270	98173	93122	96621	98821	101284	108667	118733	103052	80188	90359	82138
Observed PTs[b] (normalized)[c]	5527 (3478)	5660 (3572)	5444 (3404)	5886 (3463)	5898 (3517)	6004 (3504)	6008 (3572)	5780 (3392)	5578 (3353)	5175 (2910)	4452 (2522)	4689 (2720)
Chao1 (normalized)[c]	7925 (5967)	8092 (5999)	7908 (5797)	9270 (6431)	9151 (6383)	9723 (6339)	9382 (6094)	9177 (5734)	8510 (5744)	8668 (6121)	7092 (4988)	7542 (5481)
Shannon (normalized)[c]	9.3 (9.2)	9.3 (9.2)	9.3 (9.2)	8.0 (7.9)	8.3 (8.2)	8.3 (8.2)	8.4 (8.3)	7.6 (7.5)	8.2 (8.1)	8.0 (7.9)	6.6 (6.5)	7.6 (7.5)

Sampling Time	30 h											
**Treatment:[a]**	**C1.RNA**	**C2.RNA**	**C3.RNA**	**P1.RNA**	**P2.RNA**	**P3.RNA**	**R1.RNA**	**R2.RNA**	**R3.RNA**	**C.DNA**	**P.DNA**	**R.DNA**
Number of sequences	84481	83599	35563	114393	80012	78166	73608	69987	48891	59211	61820	63663
Observed PTs[b] (normalized)[c]	5077 (2866)	5011 (2855)	3247 (1788)	4944 (2769)	4082 (2237)	4033 (2241)	4299 (2421)	4329 (2355)	3544 (1911)	4274 (2583)	3558 (1997)	3465 (1909)
Chao1 (normalized)[c]	8266 (5806)	8117 (5742)	5378 (5302)	8014 (5075)	6927 (4930)	6730 (4713)	7128 (5208)	7330 (5539)	6063 (5242)	6557 (5439)	5838 (4707)	5739 (4589)
Shannon (normalized)[c]	7.7 (7.6)	7.9 (7.8)	7.9 (7.9)	6.7 (6.6)	6.7 (6.6)	6.7 (6.6)	7.5 (7.4)	7.4 (7.3)	7.4 (7.3)	9.3 (9.3)	7.1 (7.0)	6.2 (6.2)

[a]C, P, and R corresponds to unsupplemented control, protein, and RNA treatments, respectively. RNA or DNA samples of the three replicates were always pooled except for RNA samples at 30 h (the numbers of each treatment at 30 h [e.g., C1] indicate the respective replicate). DNA, 16S rRNA genes; RNA, 16S rRNA.

[b]Phylotypes (PTs) were clustered based on a sequence similarity cut-off of 97%.

[c]The data sets were normalized to 33,658 sequences for comparison of amplicon libraries of different sizes.

Table S9. Instrumentation utilized for analyses of organic acids and gases.

	Chromatograph		
**Parameter**	**Hewlett Packard 1090 Series II**	**Hp Hewlett 5890 Packard Series II**	**Schambeck SRI 8610C**
**Detected compounds**	Organic acids	Hydrogen	Carbon dioxide
**Column**	Rezex ROA-Organic Acids (300 x 7,8 mm; Phenomenex, Torrance, CA, USA)	Molecular sieve13X, 2 m x 1/8'' (Restek, Bellefonte, PA, USA)	Hayesep-D 2 m x 1/8'' (SRI Instruments, Earl St. Torrance, CA, USA)
**Oven temperature**	60 ℃	60 ℃	80 ℃
**Detector**	G1362A refractive index detector (RID)	Thermal conductivity detector (WLD)	Thermal conductivity detector (WLD)
**Detector temperature**	40 ℃	175 ℃	175 ℃
**Flow rate**	0.8 ml/min	20 ml/min	20 ml/min
**Injections volume**	20 µl	0.1 ml	0.1 ml
**Software**	ChemStation (Agilent Technologies, Böb-lingen, Germany)	EuroChrom Software for Windows (Ver: Basic Edition V3. 05, Wissenschaftliche Gerätebau, Berlin, Germany)	Peak simple Software (Ver: 4.20, SRI Instruments)
**Mobile phase/ Carrier gas**	4 mM H3PO4	Argon	Helium

Table S10. Summary of families of the *S. cerevisiae* lysate treatment (the most abundant taxa are displayed in Figure 2).[a]

Sampling time:	0 h		6 h		12 h		20 h	
Treatment[b]:	D	R	C.R	L.R	C.R	L.R	C.R	L.R
**Phylum, Class, Family[c]**	**Relative Abundance (%)**							
Actinobacteria,								
Acidimicrobiia,								
Acidimicrobiaceae (8)	2.1	1.2	1.7	0.6	1.5	0.6	1.3	0.7
TM214 group (4)	1.0	0.3	0.4	0.2	0.6	0.2	0.3	0.2
Unassigned *Acidimicrobiales* (6)	1.0	0.4	0.6	0.3	0.8	0.2	0.5	0.2
Actinobacteria,								
Mycobacteriaceae (2)	0.5	0.2	0.2	0.1	0.2	0.0	0.1	0.0
Nakamurellaceae (1)	0.3	0.3	0.3	0.1	0.4	0.1	0.2	0.1
Cellulomonadaceae (2)	0.1	0.5	0.6	0.3	0.7	0.2	0.5	0.2
Intrasporangiaceae (1)	0.4	0.3	0.5	0.1	0.6	0.1	0.4	0.1
Microbacteriaceae (5)	1.7	0.7	1.0	0.4	1.6	0.3	0.9	0.4
Micrococcaceae (1)	0.3	0.2	0.2	0.1	0.2	0.1	0.1	0.1
Micromonosporaceae (1)	0.1	0.1	0.1	0.0	0.1	0.0	0.1	0.0
Nocardioidaceae (7)	1.7	0.6	0.7	0.3	0.9	0.3	0.7	0.3
Propionibacteriaceae (3)	0.5	0.3	0.4	0.1	0.4	0.1	0.3	0.1
Streptomycetaceae (2)	0.6	0.4	0.3	0.1	0.4	0.1	0.2	0.1
Unassigned *Actinobacteria* (4)	2.2	1.0	1.1	0.4	1.1	0.4	0.8	0.4
Thermoleophilia,								
Gaiellaceae (2)	0.8	0.2	0.2	0.1	0.3	0.1	0.1	0.1
Unassigned *Gaiellaceae* (5)	1.9	0.3	0.3	0.1	0.5	0.1	0.3	0.1
Patulibacteraceae (1)	0.1	0.2	0.2	0.1	0.2	0.1	0.1	0.1
Solirubrobacteraceae (2)	0.2	0.4	0.4	0.1	0.5	0.1	0.3	0.1
Unassigned *Solirubrobacteraceae* (3)	1.1	0.2	0.2	0.1	0.3	0.1	0.2	0.1
***Chloroflexi**,*								
Unassigned *Chloroflexi* (3)	1.5	0.5	0.5	0.2	0.7	0.2	0.4	0.3
***Firmicutes**,*								
Bacilli,								
Bacillaceae (4)	1.5	2.0	1.3	1.9	1.6	0.4	1.0	0.5
Paenibacillaceae (1)	0.0	0.0	0.0	0.1	0.2	0.1	0.1	0.2
Clostridia,								
Clostridiaceae (10) **[CL8, CL10, CL18, CL15]**	0.0	0.0	0.1	1.0	0.6	4.3	0.7	5.9
Lachnospiraceae (5) **[CL6]**	0.0	0.0	0.0	0.0	0.0	0.5	0.0	3.4
Peptostreptococcaceae (14) **[CL2, CL5]**	0.2	0.4	4.6	36.6	12.1	38.6	17.1	27.8
***Proteobacteria**,*								
Alphaproteobacteria,								
Bradyrhizobiaceae (1)	1.2	1.8	1.7	0.6	1.2	0.4	1.0	0.4
Hyphomicrobiaceae (2)	0.2	0.2	0.3	0.1	0.3	0.1	0.2	0.1
Methylobacteriaceae (2)	0.2	0.3	0.2	0.1	0.3	0.1	0.2	0.1
Phyllobacteriaceae (1)	0.3	0.4	0.5	0.2	0.5	0.1	0.3	0.1

Sampling time:	0 h		6 h		12 h		20 h	
Treatment[b]:	D	R	C.R	L.R	C.R	L.R	C.R	L.R
**Phylum, Class, Family[c]**	**Relative Abundance (%)**							
Rhodobiaceae (1)	1.4	0.6	0.7	0.3	0.7	0.2	0.6	0.2
Xanthobacteraceae (4)	2.5	1.1	1.3	0.4	1.6	0.4	1.2	0.4
Acetobacteraceae (1)	0.0	0.2	0.1	0.1	0.1	0.0	0.1	0.1
Rhodobacteraceae (3)	0.2	0.8	0.7	0.3	0.7	0.3	0.4	0.3
Rhodospirillaceae (1)	0.0	0.1	0.1	0.0	0.1	0.0	0.1	0.0
Unassigned *Rhodospirillales* (6)	0.0	0.8	1.2	1.2	0.5	1.6	0.4	1.1
Deltaproteobacteria,								
Nitrospinaceae (3)	0.2	0.8	0.8	0.3	0.8	0.3	0.7	0.3
Sorangiineae (2)	0.1	0.3	0.3	0.1	0.4	0.1	0.2	0.1
Unassigned *Deltaproteobacteria* (2)	0.6	0.5	0.3	0.2	0.4	0.1	0.3	0.1
Gammaproteobacteria,								
Aeromonadaceae (3) **[CL7]**	5.8	3.0	0.4	10.4	0.4	8.9	0.6	7.2
Shewanellaceae (1)	0.0	0.0	0.0	0.0	0.0	0.0	0.0	0.1
Enterobacteriaceae (7**) [CL4]**	0.6	0.4	0.2	3.5	0.3	8.6	0.6	14.6
***Planctomycetes**,*								
Planctomycetacia,								
Planctomycetaceae (13)	0.8	1.1	1.6	0.8	1.3	0.8	2.0	0.8
***Tenericutes**,*								
Mollicutes,								
Mycoplasmataceae (4)	6.0	36.8	26.8	14.0	23.8	10.4	27.1	12.9
***Verrucomicrobia**,*								
Spartobacteria,								
Xiphinematobacteraceae (2)	3.3	4.2	6.5	2.5	3.2	2.5	5.1	2.5
Unassigned *Chthoniobacterales* (1)	1.1	0.2	0.2	0.1	0.1	0.1	0.1	0.1

Sampling time:	30 h							
Treatment[b]:	D.C	D.L	C1.R	C2.R	C3.R	L1.R	L2.R	L3.R
**Phylum, Class, Family[c]**	**Relative Abundance (%)**							
Actinobacteria,								
Acidimicrobiia,								
Acidimicrobiaceae (8)	1.4	1.3	2.0	1.9	1.5	0.7	1.2	0.9
TM214 group (4)	0.6	0.6	0.6	0.5	0.4	0.3	0.3	0.3
Unassigned *Acidimicrobiales* (6)	0.6	0.5	0.7	0.6	0.6	0.3	0.4	0.3
Actinobacteria,								
Mycobacteriaceae (2)	0.2	0.3	0.2	0.1	0.2	0.1	0.1	0.1
Nakamurellaceae (1)	0.1	0.2	0.4	0.4	0.3	0.3	0.2	0.1
Cellulomonadaceae (2)	0.2	0.1	0.6	0.7	0.6	0.3	0.3	0.3
Intrasporangiaceae (1)	0.3	0.3	0.5	0.4	0.4	0.2	0.2	0.2

Sampling time:	30 h							
Treatment[b]:	D.C	D.L	C1.R	C2.R	C3.R	L1.R	L2.R	L3.R
**Phylum, Class, Family[c]**	**Relative Abundance (%)**							
Microbacteriaceae (5)	0.9	1.2	1.5	1.4	1.6	1.1	0.6	0.5
Micrococcaceae (1)	0.1	0.2	0.2	0.2	0.1	0.1	0.1	0.1
Micromonosporaceae (1)	0.1	0.1	0.1	0.1	0.1	0.0	0.0	0.0
Nocardioidaceae (7)	1.0	1.0	0.9	0.9	0.7	0.3	0.4	0.3
Propionibacteriaceae (3)	0.2	0.2	0.4	0.4	0.4	0.2	0.2	0.2
Streptomycetaceae (2)	0.3	0.4	0.3	0.3	0.4	0.3	0.2	0.2
Unassigned *Actinobacteria* (4)	1.0	1.0	1.0	0.9	0.7	0.4	0.5	0.4
Thermoleophilia,								
Gaiellaceae (2)	0.4	0.4	0.2	0.2	0.2	0.1	0.1	0.1
Unassigned *Gaiellaceae* (5)	0.8	0.9	0.4	0.4	0.3	0.2	0.2	0.2
Patulibacteraceae (1)	0.1	0.2	0.1	0.1	0.2	0.2	0.1	0.1
Solirubrobacteraceae (2)	0.2	0.2	0.4	0.4	0.3	0.2	0.2	0.1
Unassigned *Solirubrobacteraceae* (3)	0.4	0.5	0.3	0.2	0.3	0.1	0.1	0.1
***Chloroflexi**,*								
Unassigned *Chloroflexi* (3)	0.9	0.8	0.6	0.6	0.5	0.3	0.4	0.3
***Firmicutes**,*								
Bacilli,								
Bacillaceae (4)	3.3	1.3	1.5	1.4	1.8	1.1	0.8	0.7
Paenibacillaceae (1)	0.5	0.1	0.1	0.2	0.1	0.1	0.1	0.1
Clostridia,								
Clostridiaceae (10) **[CL8, CL10, CL18, CL15]**	8.5	10.6	0.9	1.1	1.1	11.4	8.6	10.9
Lachnospiraceae (5) **[CL6]**	0.5	1.3	0.1	0.1	0.1	10.8	8.7	6.4
Peptostreptococcaceae (14) **[CL2, CL5]**	16.5	29.4	7.0	9.9	11.2	20.3	18.7	23.3
***Proteobacteria**,*								
Alphaproteobacteria,								
Bradyrhizobiaceae (1)	0.5	0.6	0.9	0.6	0.8	0.3	0.4	0.5
Hyphomicrobiaceae (2)	0.1	0.2	0.3	0.3	0.2	0.1	0.2	0.1
Methylobacteriaceae (2)	0.1	0.1	0.2	0.2	0.2	0.1	0.1	0.1
Phyllobacteriaceae (1)	0.1	0.1	0.2	0.2	0.3	0.1	0.1	0.1
Rhodobiaceae (1)	0.7	0.8	0.7	0.6	0.7	0.3	0.5	0.3
Xanthobacteraceae (4)	1.0	1.1	1.4	1.6	1.1	0.8	0.6	0.5
Acetobacteraceae (1)	0.0	0.0	0.1	0.1	0.1	0.1	0.1	0.0
Rhodobacteraceae (3)	0.1	0.1	0.4	0.4	0.4	0.3	0.3	0.2
Rhodospirillaceae (1)	0.0	0.0	0.1	0.1	0.1	0.0	0.1	0.0
Unassigned *Rhodospirillales* (6)	0.5	0.4	0.2	1.5	1.4	0.7	0.6	0.7
Deltaproteobacteria,								
Nitrospinaceae (3)	0.1	0.1	0.5	0.5	0.2	0.1	0.2	0.2
Sorangiineae (2)	0.1	0.1	0.4	0.5	0.3	0.2	0.2	0.1
Unassigned *Deltaproteobacteria* (2)	0.2	0.1	0.2	0.2	0.1	0.0	0.0	0.0
Gammaproteobacteria,								
Aeromonadaceae (3) [CL7]	4.8	0.2	0.1	0.2	0.1	1.3	0.9	1.2

Sampling time:	30 h							
Treatment[b]:	D.C	D.L	C1.R	C2.R	C3.R	L1.R	L2.R	L3.R
**Phylum, Class, Family[c]**	**Relative Abundance (%)**							
Shewanellaceae (1)	2.0	0.0	0.0	0.0	0.0	0.0	0.0	0.0
Enterobacteriaceae (7) **[CL4]**	1.4	12.3	0.3	0.3	0.2	10.2	9.4	13.1
Planctomycetes,								
Planctomycetacia,								
Planctomycetaceae (13)	0.7	0.4	2.9	2.6	2.0	0.8	1.4	1.2
Tenericutes,								
Mollicutes,								
Mycoplasmataceae (4)	8.7	2.1	23.8	28.0	29.0	12.8	12.9	11.0
Verrucomicrobia,								
Spartobacteria,								
Xiphinematobacteraceae (2)	2.7	1.8	5.9	5.2	4.9	2.4	4.7	4.6
Unassigned *Chthoniobacterales* (1)	0.9	0.2	0.1	0.1	0.1	0.0	0.0	0.0

[a]Listed are families that had at least one phylotype with ≥ 1,000 reads.

[b]Abbreviations: D, 16S rRNA genes; R, 16S rRNA; C, control treatment; L, *S.cerevisiae* lysate treatment. The numbers of each treatment at 30 h (e.g., C1) indicate the respective replicates.

[c]The number of phylotypes with ≥ 1,000 reads are shown in parenthesis. Phylotypes that responded to lysate amendment are bold and in brackets (see Figure 5B for detailed relative abundancies of responsive phylotypes).

Table S11. Summary of families of the protein and RNA treatments (the most abundant taxa are displayed in Figure 4).[a]

**Sampling time:**	**0 h**						**10 h**			**20 h**		
**Treatment[b]:**	**p.D**	**p.R**	**C.D**	**C.R**	**r.D**	**r.R**	**p.R**	**C.R**	**r.R**	**p.R**	**C.R**	**r.R**
**Phylum, Class, Family[c]**	**Relative Abundance (%)**											
Actinobacteria,												
Acidimicrobiia,												
Acidimicrobiaceae (7)	1.2	0.8	1.2	1.0	1.2	1.0	0.7	1.1	1.0	0.5	1.1	0.7
Unassigned *Acidimicrobiales* (7)	1.3	0.8	1.3	0.7	1.3	0.8	0.7	1.0	0.8	0.5	0.9	0.7
Actinobacteria,												
Mycobacteriaceae (2)	0.4	0.1	0.4	0.1	0.4	0.1	0.0	0.0	0.0	0.0	0.1	0.0
Unassigned Corynebacteriales (1)	0.1	0.1	0.1	0.1	0.1	0.1	0.0	0.1	0.0	0.0	0.1	0.0
Cellulomonadaceae (1)	0.1	0.3	0.1	0.3	0.1	0.3	0.3	0.4	0.3	0.2	0.4	0.3
Intrasporangiaceae (1)	0.4	0.2	0.4	0.3	0.4	0.2	0.3	0.3	0.3	0.2	0.3	0.3
Microbacteriaceae (5)	1.0	1.3	1.0	1.4	0.9	1.3	0.9	1.3	0.8	0.6	0.9	0.6
Micrococcaceae (1)	0.2	0.1	0.2	0.1	0.2	0.1	0.1	0.1	0.1	0.1	0.1	0.1
Micromonosporaceae (2)	0.3	0.2	0.3	0.2	0.3	0.2	0.2	0.3	0.2	0.1	0.2	0.2
Nakamurellaceae (1)	0.1	0.2	0.1	0.2	0.1	0.2	0.2	0.2	0.2	0.1	0.2	0.2
Nocardioidaceae (4)	0.8	0.4	0.8	0.4	0.8	0.4	0.3	0.5	0.4	0.2	0.4	0.3
Propionibacteriaceae (3)	1.0	0.4	0.9	0.4	0.9	0.5	0.3	0.5	0.4	0.3	0.5	0.3
Pseudonocardiaceae (3)	0.6	0.5	0.6	0.5	0.6	0.6	0.6	0.7	0.6	0.3	0.6	0.5
Streptomycetaceae (2)	0.5	0.4	0.5	0.4	0.4	0.3	0.3	0.3	0.3	0.2	0.3	0.3
DA023 group (13)	2.3	1.8	2.2	1.5	2.2	1.5	1.1	1.4	1.2	0.5	0.9	0.9
Unassigned *Holophagae* (1)	0.1	0.0	0.1	0.0	0.1	0.1	0.0	0.1	0.0	0.0	0.0	0.0
Thermoleophilia,												
Gaiellaceae (1)	0.5	0.1	0.5	0.1	0.5	0.2	0.2	0.2	0.2	0.1	0.2	0.2
Unassigned *Gaiellales* (8)	2.3	0.5	2.2	0.5	2.2	0.5	0.5	0.7	0.6	0.3	0.6	0.5
Patulibacteraceae (1)	0.1	0.1	0.2	0.1	0.2	0.1	0.1	0.1	0.1	0.1	0.1	0.1
Solirubrobacteraceae (4)	0.8	1.0	0.9	1.0	0.9	1.0	0.8	1.2	0.9	0.5	1.1	0.8
Unassigned *Solirubrobacterales* (6)	1.2	0.3	1.3	0.3	1.3	0.3	0.2	0.3	0.3	0.2	0.3	0.2
Unassigned *Actinobacteria* (5)	1.6	0.6	1.7	0.6	1.6	0.7	0.6	0.9	0.7	0.4	0.7	0.6
***Chloroflexi**,*												
Caldilineae,												
Caldilineaceae (1)	0.1	0.1	0.1	0.1	0.1	0.1	0.1	0.1	0.1	0.1	0.1	0.1
Chloroflexia,												
Chloroflexaceae (1)	0.1	0.1	0.1	0.1	0.1	0.1	0.1	0.1	0.1	0.0	0.1	0.1
KD4-96 group (4)	2.6	0.9	2.7	0.9	2.8	0.9	0.7	1.0	0.7	0.5	0.7	0.6
Unassigned *Thermomicrobia* (2)	0.3	0.1	0.4	0.1	0.4	0.1	0.1	0.1	0.1	0.0	0.1	0.1
Unassigned *Chlorofexi* (3)	0.5	0.2	0.6	0.2	0.5	0.2	0.1	0.2	0.2	0.1	0.2	0.1
Firmicutes												
Bacilli												
Bacillaceae (6)	0.6	0.6	0.6	0.6	0.6	0.8	0.4	0.6	1.1	0.3	0.5	0.8
Clostridia												
Clostridiaceae (9) **[PR7, PR12]**	0.2	0.3	0.2	0.2	0.2	0.2	3.4	0.5	0.5	7.8	0.8	0.6

Sampling time:	0 h						10 h			20 h		
Treatment[b]:	p.D	p.R	C.D	C.R	r.D	r.R	p.R	C.R	r.R	p.R	C.R	r.R
**Phylum, Class, Family[c]**	**Relative Abundance (%)**											
Lachnospiraceae (1)	0.0	0.0	0.0	0.0	0.0	0.0	0.0	0.0	0.0	0.0	0.0	0.1
Peptostreptococcaceae (4) **[PR2, PR8]**	0.2	0.2	0.2	0.1	0.1	0.1	17.3	1.4	0.8	25.8	4.1	0.9
Unassigned *Clostridiales* (1)	0.0	0.0	0.0	0.0	0.0	0.0	0.0	0.0	0.0	0.1	0.1	0.0
***Fusobacteria**,*												
Fusobacteria,												
Fusobacteriaceae (1) **[PR6]**	0.0	0.0	0.1	0.0	0.0	0.0	0.5	0.1	0.2	4.0	0.8	0.1
***Nitrospirae**,*												
Nitrospira,												
Nitrospiraceae (1)	0.0	0.1	0.1	0.1	0.1	0.1	0.1	0.1	0.1	0.0	0.0	0.0
Unassigned *Nitrospirales* (3)	0.5	0.3	0.5	0.3	0.5	0.3	0.2	0.3	0.3	0.2	0.3	0.4
Planctomycetes												
Planctomycetacia,												
Planctomycetaceae (64)	5.9	8.8	6.1	9.0	6.8	9.1	7.0	9.3	9.5	5.3	10.2	10.3
Unassigned *Phycisphaerae* (7)	1.2	1.0	1.0	0.8	1.3	0.9	0.6	0.7	1.0	0.3	0.7	0.8
Unassigned *Planctomycetales* (1)	0.1	0.1	0.1	0.1	0.1	0.1	0.0	0.0	0.1	0.0	0.1	0.0
***Proteobacteria**,*												
Alphaproteobacteria,												
Bradyrhizobiaceae (1)	0.7	1.2	0.6	1.1	0.6	1.2	1.0	1.3	1.0	0.7	1.2	0.7
Hyphomicrobiaceae (2)	0.2	0.2	0.2	0.2	0.2	0.2	0.2	0.2	0.2	0.1	0.2	0.1
Methylobacteriaceae (2)	0.2	0.2	0.2	0.2	0.2	0.3	0.2	0.3	0.2	0.2	0.3	0.2
Phyllobacteriaceae (1)	0.2	0.3	0.2	0.3	0.2	0.3	0.3	0.4	0.3	0.2	0.3	0.2
Rhodobiaceae (1)	1.1	0.4	1.0	0.4	1.0	0.4	0.3	0.4	0.3	0.2	0.4	0.2
Xanthobacteraceae (7)	2.4	1.4	2.4	1.2	2.2	1.4	1.1	1.5	1.2	0.8	1.5	0.9
Unassigned *Rhizobiales* (1)	0.1	0.1	0.0	0.1	0.1	0.1	0.1	0.1	0.1	0.0	0.1	0.0
Rhodobacteraceae (1)	0.1	0.1	0.1	0.1	0.1	0.1	0.1	0.1	0.1	0.1	0.1	0.1
Rhodospirillaceae (7)	0.6	1.3	0.6	1.2	0.6	1.3	1.3	1.7	1.4	0.8	1.5	1.2
Unassigned *Rhodospirillales* (4)	0.4	0.6	0.5	0.5	0.5	0.5	0.6	0.6	0.6	0.3	0.6	0.5
Betaproteobacteria,												
Alcaligenaceae (1)	0.1	0.1	0.1	0.1	0.1	0.1	0.1	0.1	0.1	0.1	0.1	0.1
Comamonadaceae (1)	0.0	0.1	0.0	0.1	0.0	0.1	0.1	0.1	0.1	0.1	0.1	0.1
Deltaproteobacteria,												
Nitrospinaceae (2)	0.1	0.7	0.1	0.6	0.1	0.6	0.6	0.6	0.6	0.3	0.5	0.5
Cystobacteraceae (1)	0.1	0.1	0.1	0.1	0.1	0.1	0.1	0.1	0.2	0.0	0.1	0.1
Nannocystineae (1)	0.0	0.1	0.0	0.1	0.0	0.1	0.0	0.1	0.0	0.0	0.1	0.1
Sorangiineae (1)	0.0	0.1	0.0	0.1	0.0	0.1	0.1	0.1	0.1	0.0	0.1	0.1
Unassigned *Deltaproteobacteria* (2)	0.5	0.4	0.5	0.4	0.5	0.4	0.3	0.5	0.4	0.3	0.4	0.3
Gammaproteobacteria,												
Aeromonadaceae (4) **[PR3]**	0.4	0.1	0.3	0.1	0.3	0.8	1.3	0.3	12.0	0.7	0.3	25.0
Shewanellaceae (1)	0.0	0.0	0.0	0.0	0.0	0.0	0.0	0.0	0.0	0.0	0.0	0.0
Enterobacteriaceae (3) **[PR33]**	0.1	0.3	0.1	0.1	0.1	0.2	0.6	0.1	0.4	0.7	0.2	0.7
Sinobacteraceae (1)	0.1	0.1	0.1	0.1	0.1	0.1	0.1	0.1	0.1	0.0	0.1	0.0

Sampling time:	0 h						10 h			20 h		
Treatment[b]:	p.D	p.R	C.D	C.R	r.D	r.R	p.R	C.R	r.R	p.R	C.R	r.R
**Phylum, Class, Family[c]**	Relative Abundance (%)											
Tenericutes,												
Mollicutes,												
Mycoplasmataceae (3)	13.2	28.8	13.0	32.3	11.0	28.1	20.3	25.6	20.6	20.0	28.1	15.3
Unassigned *Mollicutes* (2)	0.2	0.3	0.2	0.3	0.2	0.3	0.2	0.2	0.2	0.2	0.2	0.1
Verrucomicrobia,												
Spartobacteria,												
Chthoniobacteraceae (1)	0.1	0.2	0.2	0.2	0.2	0.2	0.1	0.1	0.1	0.1	0.1	0.0
Xiphinematobacteraceae (2)	4.5	5.0	4.9	4.4	5.5	5.3	5.0	5.6	4.2	3.7	4.6	2.8
Unassigned *Chthoniobacterales* (8)	3.7	0.7	4.3	0.8	4.6	0.8	0.5	0.6	0.5	0.3	0.5	0.4
Verrucomicrobiae,												
Verrucomicrobiaceae (1)	0.2	0.1	0.1	0.1	0.2	0.1	0.0	0.1	0.0	0.0	0.0	0.0

Sampling time:	30 h											
Treatment[b]:	p.D	p1.R	p2.R	p3.R	C.D	C1.R	C2.R	C3.R	r.D	r1.R	r2.R	r3.R
**Phylum, Class, Family[c]**	Relative Abundance (%)[b]											
Actinobacteria,												
Acidimicrobiia,												
Acidimicrobiaceae (7)	0.6	0.6	0.6	0.6	1.2	1.0	1.0	0.8	0.6	0.8	0.7	1.1
Unassigned *Acidimicrobiales* (7)	0.5	0.4	0.4	0.5	1.3	0.9	0.8	0.7	0.5	0.7	0.6	0.8
Actinobacteria,												
Mycobacteriaceae (2)	0.3	0.0	0.0	0.0	0.3	0.1	0.1	0.1	0.2	0.1	0.0	0.0
Unassigned Corynebacteriales (1)	0.0	0.0	0.0	0.0	0.1	0.0	0.1	0.0	0.0	0.0	0.0	0.0
Cellulomonadaceae (1)	0.1	0.2	0.2	0.2	0.3	0.4	0.4	0.3	0.1	0.3	0.3	0.5
Intrasporangiaceae (1)	0.2	0.1	0.2	0.2	0.4	0.3	0.3	0.2	0.2	0.2	0.2	0.2
Microbacteriaceae (5)	0.4	0.5	0.6	0.6	0.9	1.1	0.8	0.8	0.4	1.0	0.7	1.2
Micrococcaceae (1)	0.1	0.1	0.0	0.1	0.1	0.1	0.1	0.1	0.1	0.1	0.1	0.1
Micromonosporaceae (2)	0.1	0.1	0.1	0.2	0.3	0.2	0.3	0.2	0.1	0.2	0.2	0.2
Nakamurellaceae (1)	0.1	0.1	0.1	0.1	0.1	0.1	0.2	0.2	0.0	0.2	0.1	0.2
Nocardioidaceae (4)	0.3	0.2	0.2	0.2	0.7	0.3	0.5	0.3	0.3	0.3	0.3	0.4
Propionibacteriaceae (3)	0.4	0.3	0.3	0.3	1.0	0.4	0.5	0.4	0.4	0.4	0.3	0.5
Pseudonocardiaceae (3)	0.2	0.3	0.4	0.3	0.5	0.5	0.6	0.5	0.3	0.5	0.5	0.7
Streptomycetaceae (2)	0.2	0.1	0.2	0.2	0.5	0.3	0.4	0.3	0.2	0.3	0.3	0.4
DA023 group (13)	0.8	0.2	0.4	0.4	1.8	0.7	0.8	0.4	0.6	0.6	0.7	0.6
Unassigned *Holophagae* (1)	0.1	0.0	0.0	0.0	0.2	0.1	0.0	0.0	0.1	0.0	0.0	0.1
Thermoleophilia,												
Gaiellaceae (1)	0.2	0.1	0.1	0.1	0.5	0.2	0.2	0.2	0.2	0.2	0.1	0.2
Unassigned *Gaiellales* (8)	1.0	0.3	0.3	0.4	2.4	0.7	0.6	0.6	1.0	0.6	0.5	0.7
Patulibacteraceae (1)	0.1	0.0	0.0	0.1	0.2	0.1	0.1	0.1	0.1	0.1	0.1	0.1
Solirubrobacteraceae (4)	0.3	0.4	0.5	0.5	0.8	1.0	1.0	0.8	0.3	0.8	0.7	1.1

Sampling time:	30 h											
Treatment[b]:	p.D	p1.R	p2.R	p3.R	C.D	C1.R	C2.R	C3.R	r.D	r1.R	r2.R	r3.R
**Phylum, Class, Family[c]**	**Relative Abundance (%)[b]**											
Unassigned *Solirubrobacterales* (6)	0.5	0.2	0.2	0.2	1.1	0.3	0.3	0.3	0.5	0.2	0.2	0.3
Unassigned *Actinobacteria* (5)	0.7	0.4	0.4	0.4	1.6	0.7	0.8	0.7	0.7	0.6	0.5	0.8
Chloroflexi,												
Caldilineae,												
Caldilineaceae (1)	0.0	0.1	0.1	0.1	0.1	0.1	0.1	0.1	0.0	0.1	0.1	0.1
Chloroflexia,												
Chloroflexaceae (1)	0.0	0.0	0.0	0.0	0.1	0.0	0.0	0.0	0.1	0.1	0.0	0.0
KD4-96 group (4)	1.1	0.4	0.4	0.5	2.9	0.9	0.7	0.6	1.1	0.7	0.7	0.9
Unassigned *Thermomicrobia* (2)	0.2	0.0	0.1	0.1	0.4	0.1	0.1	0.1	0.2	0.1	0.1	0.1
Unassigned *Chlorofexi* (3)	0.2	0.1	0.1	0.1	0.6	0.2	0.2	0.1	0.2	0.1	0.2	0.2
Firmicutes												
Bacilli												
Bacillaceae (6)	1.7	0.4	0.3	0.4	1.0	0.6	0.7	0.7	1.1	1.2	1.2	1.8
Clostridia												
Clostridiaceae (9) **[PR7, PR12]**	13.8	11.1	13.3	11.9	1.5	1.4	1.1	1.3	0.8	1.8	1.7	3.0
Lachnospiraceae (1)	0.3	0.3	0.2	0.2	0.2	0.1	0.1	0.1	0.7	1.1	1.1	2.2
Peptostreptococcaceae (4) **[PR2, PR8]**	18.2	18.4	20.6	23.1	3.4	4.1	3.6	3.5	1.3	2.2	2.4	4.5
Unassigned *Clostridiales* (1)	0.2	0.5	0.4	0.6	0.1	0.3	0.4	0.4	0.0	0.0	0.0	0.0
Fusobacteria,												
Fusobacteria,												
Fusobacteriaceae (1) **[PR6]**	11.5	10.5	7.9	6.3	1.4	0.6	1.6	0.5	0.6	0.2	0.2	0.2
Nitrospirae,												
Nitrospira,												
Nitrospiraceae (1)	0.0	0.0	0.0	0.0	0.1	0.0	0.0	0.0	0.0	0.1	0.1	0.1
Unassigned *Nitrospirales* (3)	0.2	0.1	0.1	0.2	0.6	0.2	0.3	0.2	0.2	0.3	0.2	0.3
Planctomycetes												
Planctomycetacia,												
Planctomycetaceae (64)	3.2	5.7	5.6	6.1	6.6	9.2	9.5	6.4	3.4	8.1	8.3	10.6
Unassigned *Phycisphaerae* (7)	0.6	0.3	0.3	0.3	1.0	0.5	0.5	0.3	0.6	0.5	0.5	0.5
Unassigned *Planctomycetales* (1)	0.0	0.0	0.0	0.0	0.1	0.1	0.1	0.0	0.0	0.0	0.0	0.0
Proteobacteria,												
Alphaproteobacteria,												
Bradyrhizobiaceae (1)	0.3	0.5	0.6	0.6	0.6	1.0	1.2	0.9	0.2	0.6	0.8	1.0
Hyphomicrobiaceae (2)	0.1	0.1	0.1	0.1	0.1	0.2	0.2	0.2	0.1	0.1	0.1	0.2
Methylobacteriaceae (2)	0.1	0.1	0.1	0.1	0.2	0.2	0.3	0.2	0.1	0.2	0.2	0.2
Phyllobacteriaceae (1)	0.1	0.2	0.2	0.2	0.2	0.3	0.3	0.2	0.1	0.2	0.2	0.3
Rhodobiaceae (1)	0.4	0.3	0.2	0.2	0.9	0.5	0.5	0.3	0.3	0.2	0.2	0.4
Xanthobacteraceae (7)	0.9	0.7	0.7	0.8	2.1	1.5	1.6	1.2	0.8	0.9	0.9	1.2
Unassigned *Rhizobiales* (1)	0.0	0.0	0.0	0.0	0.1	0.1	0.1	0.1	0.0	0.1	0.1	0.1
Rhodobacteraceae (1)	0.0	0.1	0.1	0.0	0.1	0.1	0.1	0.1	0.0	0.1	0.1	0.1
Rhodospirillaceae (7)	0.3	0.8	0.8	0.8	0.6	1.2	1.4	1.1	0.3	1.1	1.0	1.6

Sampling time:	30 h											
Treatment[b]:	p.D	p1.R	p2.R	p3.R	C.D	C1.R	C2.R	C3.R	r.D	r1.R	r2.R	r3.R
**Phylum, Class, Family[c]**	**Relative Abundance (%)[b]**											
Unassigned *Rhodospirillales* (4)	0.2	0.3	0.4	0.3	0.5	0.5	0.6	0.4	0.2	0.5	0.4	0.5
Betaproteobacteria,												
Alcaligenaceae (1)	0.1	0.1	0.1	0.0	0.2	0.1	0.1	0.1	0.1	0.1	0.0	0.1
Comamonadaceae (1)	0.0	0.0	0.0	0.0	0.1	0.1	0.1	0.0	0.0	0.1	0.1	0.1
Deltaproteobacteria,												
Nitrospinaceae (2)	0.1	0.2	0.3	0.2	0.1	0.3	0.5	0.3	0.1	0.4	0.5	0.5
Cystobacteraceae (1)	0.0	0.0	0.0	0.0	0.1	0.1	0.1	0.0	0.0	0.1	0.1	0.1
Nannocystineae (1)	0.0	0.0	0.0	0.0	0.0	0.1	0.1	0.0	0.0	0.0	0.0	0.1
Sorangiineae (1)	0.0	0.0	0.0	0.0	0.0	0.1	0.1	0.1	0.0	0.1	0.0	0.1
Unassigned *Deltaproteobacteria* (2)	0.2	0.2	0.2	0.2	0.4	0.3	0.3	0.3	0.2	0.3	0.3	0.4
Gammaproteobacteria,												
Aeromonadaceae (4) **[PR3]**	4.4	0.3	0.4	0.3	2.2	0.1	0.3	0.1	44.5	17.9	20.7	22.2
Shewanellaceae (1)	0.7	0.0	0.0	0.0	1.1	0.0	0.0	0.0	0.5	0.2	0.2	0.2
Enterobacteriaceae (3) **[PR33]**	0.8	0.4	0.4	0.3	0.2	0.1	0.1	0.1	4.0	2.3	2.6	3.4
Sinobacteraceae (1)	0.0	0.0	0.0	0.0	0.1	0.1	0.1	0.0	0.0	0.0	0.0	0.0
***Tenericutes**,*												
Mollicutes,												
Mycoplasmataceae (3)	6.8	18.0	15.7	16.2	7.8	32.3	28.4	19.3	6.5	21.6	17.7	29.5
Unassigned *Mollicutes* (2)	0.1	0.2	0.2	0.2	0.1	0.3	0.3	0.2	0.1	0.2	0.2	0.3
***Verrucomicrobia**,*												
Spartobacteria,												
Chthoniobacteraceae (1)	0.0	0.0	0.0	0.0	0.1	0.1	0.1	0.0	0.1	0.0	0.0	0.1
Xiphinematobacteraceae (2)	2.8	4.6	4.5	3.7	4.3	4.4	5.2	3.9	3.0	3.0	5.1	6.4
Unassigned *Chthoniobacterales* (8)	2.1	0.3	0.3	0.2	3.4	0.6	0.6	0.3	1.8	0.4	0.3	0.5
Verrucomicrobiae,												
Verrucomicrobiaceae (1)	0.0	0.0	0.0	0.0	0.1	0.0	0.0	0.0	0.0	0.0	0.0	0.0

[a]Listed are families that had at least one phylotype with ≥ 1,000 reads.

[b]Abbreviations: D, 16S rRNA genes; R, 16S rRNA; C, control treatment; r, RNA treatment; p, protein treatment. The numbers of each treatment at 30 h (e.g., C1) indicate the respective replicates.

[c]The number of phylotypes with ≥ 1,000 reads are shown in parenthesis. Phylotypes that responded to protein or RNA amendment are bold and in brackets (see Figure 5B for detailed relative abundancies of responsive phylotypes).
